# Rational Design of Novel Inhibitors of α-Glucosidase: An Application of Quantitative Structure Activity Relationship and Structure-Based Virtual Screening

**DOI:** 10.3390/ph14050482

**Published:** 2021-05-19

**Authors:** Sobia Ahsan Halim, Sumaira Jabeen, Ajmal Khan, Ahmed Al-Harrasi

**Affiliations:** 1 Natural and Medical Sciences Research Center, University of Nizwa, P.O. Box 33, Birkat Al Mauz 616, Nizwa, Oman; sobia_halim@unizwa.edu.om; 2 Department of Chemistry, Government Post-Graduate College for Women, Shadbagh, Lahore 54950, Pakistan; sumairabhatti23@gmail.com

**Keywords:** α-Glucosidase, QSAR modeling, homology modeling, molecular docking, ADMET profiling

## Abstract

α-Glucosidase is considered a prime drug target for Diabetes Mellitus and its inhibitors are used to delay carbohydrate digestion for the treatment of diabetes mellitus. With the aim to design α-glucosidase inhibitors with novel chemical scaffolds, three folds ligand and structure based virtual screening was applied. Initially linear quantitative structure activity relationship (QSAR) model was developed by a molecular operating environment (MOE) using a training set of thirty-two known inhibitors, which showed good correlation coefficient (r^2^ = 0.88), low root mean square error (RMSE = 0.23), and cross-validated correlation coefficient r^2^ (q^2^ = 0.71 and RMSE = 0.31). The model was validated by predicting the biological activities of the test set which depicted r^2^ value of 0.82, indicating the robustness of the model. For virtual screening, compounds were retrieved from zinc is not commercial (ZINC) database and screened by molecular docking. The best docked compounds were chosen to assess their pharmacokinetic behavior. Later, the α-glucosidase inhibitory potential of the selected compounds was predicted by their mode of binding interactions. The predicted pharmacokinetic profile, docking scores and protein-ligand interactions revealed that eight compounds preferentially target the catalytic site of α-glucosidase thus exhibit potential α-glucosidase inhibition *in silico*. The α-glucosidase inhibitory activities of those Hits were predicted by QSAR model, which reflect good inhibitory activities of these compounds. These results serve as a guidelines for the rational drug design and development of potential novel anti-diabetic agents.

## 1. Introduction

Diabetes mellitus (DM) is one of the most prominent metabolic, chronic and persistent diseases illustrated by hyperglycemia, which itself poses a great global health challenge [1,2]. The morbidity rate of DM is expected to exceed up to 10.4% by 2040 globally [3]. DM is linked with impaired production of insulin or due to improper response of body cells towards insulin. Several complications including hypertension, kidney failure, loss of vision, neuropathy, cardiovascular diseases (CVDs) and atherosclerosis are associated with inadequate production of insulin or due to prolonged hyperglycemia. DM is classified into Type I DM (T1DM) and Type II DM (T2DM). T1DM is also known as insulin dependent DM, which is caused by environmental, genetic and autoimmune factors. T-cell mediated autoimmune damage of *β*-cells of the pancreas is responsible for this type and illustrated by complete scarcity of insulin. One of the significant complications of this disease is ketoacidosis. Patients are susceptible to other diseases like Grave’s disease, Addison’s disease, Celiac sprain and autoimmune hepatitis [3]. T2DM is non-insulin dependent diabetes or adult onset diabetes associated with insulin resistance in peripheral tissues or relative deficiency of insulin. It is a progressive and most common type of disease. *β*-Cells are not damaged in this case; however, obesity is associated in most individuals and ketoacidosis rarely occurs. Most of the time, patients remain asymptomatic and have an enhanced risk of serious macrovascular and microvascular complications. Moreover, autooxidation of glucose and enhanced oxidative stress are also associated with T2DM. The risk of T2DM increases due to increased age, weight, improper physical activity and sedentary lifestyle [4]. In the advance stage of T2DM more complications occur as free radicals are formed, which cause insulin resistance and the dysfunction of *β*-cells.

Enhanced production of glucose is directed by α-glucosidase in the body; therefore, its level can be controlled by discovering its inhibitors [5,6]. α-Glucosidase is a lysosomal exo-glycosidase that catalyzes the breakdown of complex sugar like starch and disaccharides to glucose that are further absorbed by gut and subsequently raises postprandial hyperglycemia. Thus, α-glucosidase is the pathological hallmark of this disease. Furthermore, this enzyme has been linked in tumor metastasis in association with collagen type I and IV. In DM patients, inhibition of α-glucosidase efficiently lowers the risk of colorectal cancer and cerebrovascular events [7,8]. 

Many drugs like sulfonylureas, meglitinides, biguanides and thiazolidinediones and α-glucosidase inhibitors are most potent hypoglycemic agents [9]. Metformin, miglitol, acarbose, voglibose and glibenclamide are clinically used drugs for the management of diabetes. They are highly expensive and put a financial burden on patients, which results in huge economic loss. Their detrimental side effects also created an alarming situation like dropsy, increase in weight, resistance against drug, liver disorder, renal tumors, acute hepatitis, hepatic injury and hypoglycemia [10]. Thus, design and discovery of novel α-glucosidase inhibitors with reduced cost and lower side effects are urgently needed to control DM [11,12,13].

Computational drug designing approaches have proven effective in delivering novel chemical scaffolds in the market against several drug targets [14,15,16]. Due to our interest in finding novel leads against α-glucosidase [17,18,19], ligand and structure based virtual screening strategies were applied in this study to target α-glucosidase enzyme. The study comprises of ligand based QSAR modeling, structure-based virtual screening, pharmacokinetic profiling, and QSAR-based prediction of biological activities of new scaffolds against α-glucosidase. In-silico techniques have proven effective in the discovery of chemical agents against specific drug target with high binding affinity and specificity [15]. Several potent drugs including Captopril (Angiotensin-converting enzyme inhibitor, treats congestive heart failure and hypertension), Nelfinavir (HIV-protease inhibitor), Zanamivir (neuraminidase inhibitor of influenza viruses), Dorzolamide (carbonic anhydrase inhibitor), Cinanserin (potent inhibitor of 3C-like protease of SARS), Saquinavir, Indinavir, and Ritonavir (anti-HIV/AIDS drugs) were successfully designed by computational methods which are available on the market [20,21,22,23]. In addition, computer-aided drug designing (CADD) has delivered numerous novel anti-cancer compounds such as Erlotinib (EGFR kinase inhibitor, suppresses Non-small cell lung carcinoma and pancreatic cancer), Sorafenib (VEGFR inhibitor, treat thyroid, renal and liver cancer), Lapatinib (for the management of ERBB2-postive breast cancer), Abiraterone (inhibits androgen production to treat metastatic castration-resistant prostate cancer or hormone-refractory prostate cancer) and Crizotinib (ALK inhibitor, to treat Non-small cell lung carcinoma) [24,25,26,27,28,29,30,31,32,33,34,35,36,37,38,39]. 

Keeping the successful applications of CADD in mind, we employed both ligand and structure-based methods to design novel α-glucosidase inhibitors.

## 2. Results and Discussion

### 2.1. QSAR Modeling

**Figure 1:**
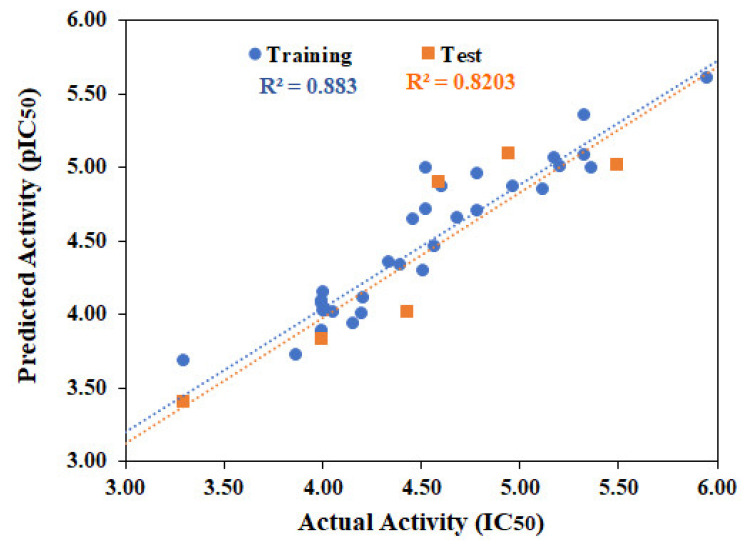
Graphical presentation of correlation between actual and predicted α-glucosidase inhibitory activities of training and test set compounds. R^2^ of training and test set are 0.883 and 0.820, respectively.

QSAR models are widely used to predict the biological activities of compounds in silico [40,41]. Molecular Operating Environment (MOE) use descriptor-based technique to develop linear QSAR model and give two values, i.e., predicted pIC_50_ and residual value. Initially 192 2D-descriptors were calculated for thirty-two known α-glucosidase inhibitors (**αGI1-αGI4, αGI6-αGI15, αGI17-αGI20, αGI22-αGI24, αGI26-αGI31, αGI33-αGI34, αGI36-αGI38**) via QuaSAR-Descriptor module and QuaSAR-contingency was used to select the appropriate descriptors. Out of 192, nine descriptors (apol, bpol, a_acc, a_heavy, logP(o/w), logS, TPSA, Weight and wienerPol) were suggested as best descriptors for our data by contingency analysis. Partial least square (PLS) regression method was applied on training set to develop model. Model adequacy was measured as the square of correlation coefficient (r^2^), root mean square error (RMSE), cross–validated r^2^ (r^2^_LOO_ or q^2^) and cross–validated RMSE. The developed model showed good correlation coefficient (r^2^ = 0.88) and low root mean square error (RMSE = 0.23), suggesting the strength of the model. Cross validation was performed using leave-one-out (LOO) cross validations scheme which resulted acceptable cross-validated correlation coefficient (q^2^ = 0.71 and RMSE = 0.31). The biological activities of the test set [six molecules (**αGI5, αGI16, αGI21, αGI25, αGI32, αGI35**)] were predicted by the model which showed r^2^ value of 0.82. The model accurately predicted the activities of test set. The chemical structures, biological activities, predicted activities and residual values of **αGI**s are summarized in Table 1. The correlation plots of training and test set are shown in Figure 1. The 2D linear regression model is shown in the equation below, which is statistically significant and explain 88% of the variability of the IC_50_ coefficient, characterized by usefulness of the model to predict the α-glucosidase inhibitory activity of test set compounds. 

One metric to measure a descriptor’s relative importance in a QSAR model is to compute the product of the magnitude of a regression coefficient times the range in values adopted by its descriptor across the training set. This metric is a measure of the variability of dependent variable of the QSAR upon the descriptor. The relative importance of descriptors is: a_heavy = 1.000, Weight = 0.773, and wienerPol = 0.718, TPSA = 0.471, a_acc = 0.424, logP(*o/w*) = 0.355, logS = 0.244, apol = 0.242, bpol = 0.207. The data shows that the weight, and wienerPol descriptors are the most important factor because of their correlation coefficient > 0.5, followed by TPSA, a_acc and logP. 

The positive coefficient of descriptor in model reflects that increasing the number of heavy (polar) atoms, hydrogen bond acceptor atoms, logP(o/w), bpol (Sum of the absolute value of the difference between atomic polarizabilities of all bonded atoms in the molecule (including implicit hydrogens) with polarizabilities) and topological polar surface area (Å^2^) will increase the activity of compounds. The descriptors a_heavy, logP(o/w), bpol and TPSA are physical properties which can be calculated from the connection table (with no dependence on conformation) of a molecule while a_acc belongs to pharmacophore feature/atom type descriptors. 

The negative coefficient of descriptors shows that decreasing some of the physical properties including apol, logS, molecular weight and one of the adjacency and distance matrix descriptors, i.e., wiener polarity number will be beneficial to enhance the activity of the compounds.

(1) $$\mathrm{pIC}50\;=\;3.39574\;-0.08965\;\left(\mathrm{apol}\right)\;+0.09305\;\left(\mathrm{bpol}\right)\;+0.49740\;\left(a\_\mathrm{acc}\right)\;+0.71639\;\left(a\_\mathrm{heavy}\right)\;+0.40979\;\left(\mathrm{logP}\left(o/w\right)\right)\;-0.37591\;\left(\mathrm{logS}\right)\;+0.02502\;\left(\mathrm{TPSA}\right)\;-0.03659\;\left(\mathrm{Weight}\right)\;-0.24818\;\left(\mathrm{wienerPol}\right)$$

r2 = 0.88, RMSE = 0.23, q2 = 0.71, RMSE(LOO) = 0.31, N(train) = 32; N (test) = 6

where apol = Sum of the atomic polarizabilities (including implicit hydrogens) with polarizabilities, bpol = Sum of the absolute value of the difference between atomic polarizabilities of all bonded atoms in the molecule (including implicit hydrogens) with polarizabilities, a_acc = hydrogen bond acceptor atoms (not counting acidic atoms but counting atoms that are both hydrogen bond donors and acceptors such as –OH), a_heavy = number of heavy atoms, logP(*o*/*w*) = Log of the octanol/water partition coefficient (including implicit hydrogens), logS = Log of the aqueous solubility (mol/L), TPSA = Polar surface area (Å^2^) calculated using group contributions to approximate the polar surface area from connection table information only, Weight = molecular weight (including implicit hydrogens) in atomic mass units with atomic weights, wienerPol = wiener polarity number (half the sum of distance matrix entries with a value of 3), r^2^ = Correlation coefficient, RMSE = Root mean square error, q^2^ = Cross-validated r^2^, RMSE_(LOO)_ = Cross-validated RMSE.

### 2.2. Structure-Based Screening of Filtered Compounds against α-Glucosidase

**Figure 2:**
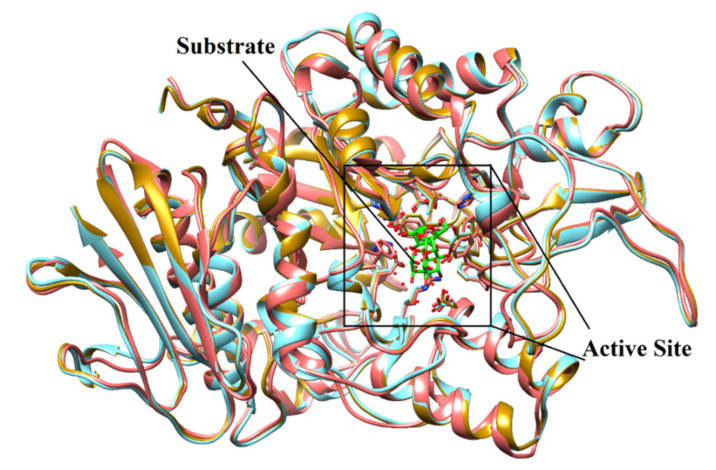
The superimposed view of model (coral ribbons) and templates [3AXH (golden ribbon) and 3A47 (Cyan ribbon)] shows the structural topology of the model is similar to its templates. The active site residues are shown in stick model. The substrate molecule (isomaltose) is depicted in green stick model.

**Figure 3:**
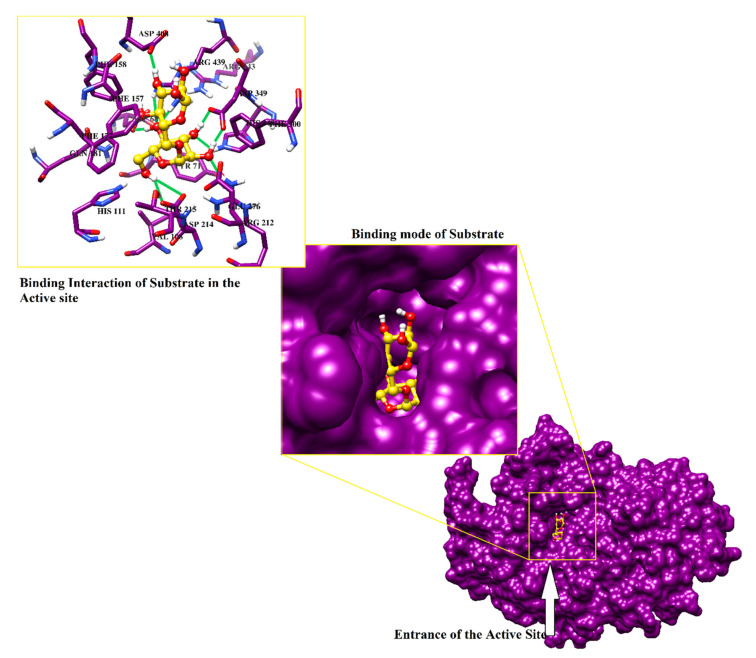
The binding mode of substrate is shown in the active site of enzyme. The binding interactions are highlighted in box. Hydrogen bonds are shown in green lines.

Previously we have generated the 3D-coordinates of *S. cerevisiae α*-glucosidase by homology modeling [17,18,19], which is composed of 579 residues. According to Ramachandran plot, the model possesses excellent stereochemical properties. Out of 579, 444 (86.7%), 63 (12.3%) and 3 (0.6%) residues lied in the most favored, additionally allowed and generously allowed regions, respectively. While two residues (0.4%) (Ala278 and Thr566) are present in disallowed regions which are not a part of active site. ERRAT showed 93.52 quality factor of the model, while in verify3D plot, 95.5% residues showed average 3D-1D score of 0.2. The stereochemical and geometric properties of the model is good and can be used in the structure-based filtration of compounds. The structural geometry and topology of the model was found to be similar to the structural topology of its template (Figure 2). The catalytic residues are conserved among *S. cerevisiae* isomaltase and *S. cerevisiae α*-glucosidase. The sequence alignment of the model and the template is shown in (Figure S1, Supporting Information). The substrate molecule (isomaltose taken from PDB ID 3AXH [22]) was manually docked to deduce the important catalytic residues of protein. Asp214, Glu276, and Asp349 constitutes the catalytic triad for substrate catalysis, where Asp214 and Glu276 serves as nucleophile and proton donor, respectively and Asp349 stabilized the transition state of substrate molecule. The lining of the active site is composed to Asp68, Tyr71, Val108, His111, Phe157, Phe158, Phe177, Gln181, Arg212, Thr215, Ala278, Phe300, Arg312, His348, Gln350, Asp408, Arg439, and Arg443 that mediates multiple hydrophilic and hydrophobic interactions with the substrate and the competitive inhibitor of α-glucosidase ‘acarbose’. Moreover, we have recognized that ten water molecules (1021,1026, 1056, 1058, 1061, 1087, 1102, 1122, 1174, and 1228) in the active site play important role in protein-substrate/protein-inhibitor bridging (Figure 3). 

For structure-based screening, ZINC database [22] was filtered according to the physicochemical properties of the substrate molecule (isomaltose) and 6609 compounds were matched with the given parameters. A dataset of 6609 compounds with 38 known inhibitors (αGIs used in QSAR modeling) was docked in the active site of *S. cerevisiae α*-glucosidase to determine their binding potential with α-glucosidase enzyme. The known inhibitors (αGIs) were embedded in the screening library to test the screening accuracy of docking method. The virtual screening accuracy was tested by using two metrics namely enrichment factor (EF) and Receiver operating characteristic-curve (ROC-curve). The analysis of EF and ROC-curve is discussed in Supporting Information. MOE showed >7, >47 and >78%EF in top 1%, 5% and 10% screened library. Additionally, ROC-curve shows area under the curve (AUC) value of 0.94, reflecting good virtual screening performance of MOE. The ROC-curve and the correlation of inhibitory activities of known inhibitors with their docking score is displayed in Figures S2 and S3, Supporting Information.

The docked library was ranked according to the docking score and top 10% of the screened compounds (>600) were selected for post-docking filtration. Based on docking ranks, scores and calculated protein-ligand interaction fingerprints (PLIF), 202 compounds were considered ‘best’ and their pharmacokinetics (absorption, distribution, metabolism, excretion and toxicity) profiling was carried out by computational tools [SwissADME (http://www.swissadme.ch/ accessed on 15 December 2020) and admetSAR (http://lmmd.ecust.edu.cn/admetsar2 accessed on 15 December 2020)].

### 2.3. ADMET Analysis of Selected Hits

The top ranked docked compounds (202 best hits) were selected for their ADMET prediction which showed that 142/202 compounds exhibited acceptable pharmacokinetic properties (named as compounds **1**–**142**, Table S1, Supporting Information). Those compounds do not penetrate blood brain barrier (BBB), display high human intestinal absorption (HIA), revealed no AMES toxicity and carcinogenicity. The calculated oral toxicity showed that these compounds fall in category III of acute oral toxicity (LD_50_ = >500 mg/kg to <5000 mg/kg), indicating that the compounds are non-toxic. The predicted RAT acute toxicity reflect that the compounds show low in vivo toxicity up to the dose of ≥2 mol/kg. The synthetic accessibility (SA) values of these compounds are also ≤5 indicating that these compounds are easily synthesizable. 

### 2.4. Protein-Ligand Interaction Analysis of 142 Compounds

**Figure 4:**
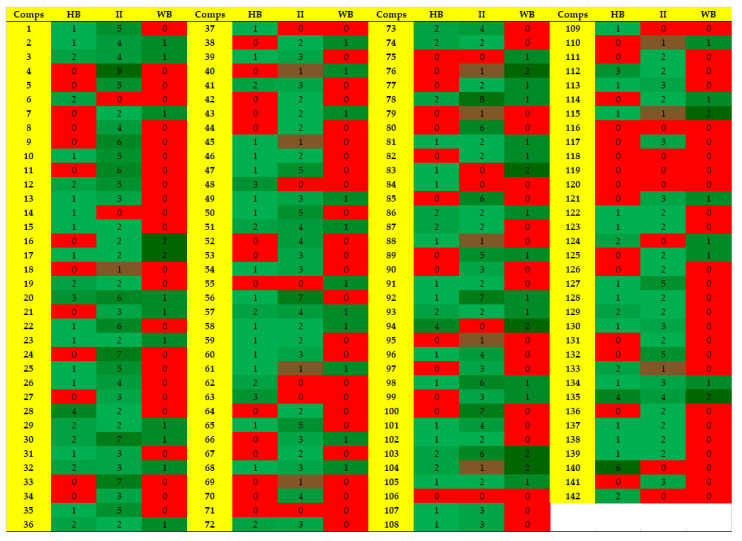
The predicted interaction pattern of compounds **1**–**142.** The binding interactions are shown in 3-color-scale (green through red colour scheme) where red and green indicates least and high number of bonds, respectively, HB = Hydrogen bonds, II = ionic interactions, WB = water bridging.

According to ADMET profile, compounds **1**–**142** were retrieved as good inhibitors. The modes of binding of all those (142) compounds were analyzed in the active site of α-glucosidase to select the potential inhibitors of α-glucosidase. The compounds were sorted on the basis of higher number of hydrogen bonding pattern within the active site, and compounds with number of hydrogen bonds ≥ 3, were selected. Subsequently eight molecules (**20, 28, 48, 63, 94, 112, 135 and 140**) were retrieved as ‘Hits’ which specifically interacted with one or two residues of catalytic triad (Asp214-Glu276-Asp349). Therefore, based on docking scores and binding interactions, those ‘Hits’ were considered as most active inhibitors. The predicted protein-ligand interactions of 142 compounds are shown in Figure 4. The docking ranks, scores, and molecular interactions of each compound are summarized in Table S2 (Supporting Information).

### 2.5. The Binding Potential of High Active Hits

The analysis of binding interactions reflect that eight compounds (**20, 28, 48, 63, 94, 112, 135** and **140**) can act as potential inhibitors of α-glucosidase enzyme. Those compounds exhibited good binding interactions within the active site of enzyme. The docked view of compound **20** showed that Glu276 and Asp214 of catalytic triad (CT) and Thr215 formed hydrogen bonds with the amino group of the compound. Moreover, the side chains of Asp68, Asp214 and Glu276 mediated ionic interaction with the amino and the nitrile groups of the compound. Additionally, a water molecule was involved in the protein-ligand bridging. These interactions are responsible for the higher binding affinity of the compound [docking score (DS) = −18.35 kcal/mol and docking rank (DR) = 34). The triazolopyrimidine ring of compound **28** (DS= −18.04 kcal/mol, DR = 46) interacted with the side chain of Asp349 of CT, while the hydroxyl group of the compound formed H-bonds with the side chains of Asp214 and Arg212. Moreover, Asp349 and Phe157 provide ionic and π-π interactions to the compound, respectively. The morpholine ring of **48** (DR = 77, and DS = −17.50 kcal/mol) interacted with Asp214 and Arg439 through H-bonds, while Asp214 also mediated ionic interaction with the compound. The amino group of the compound **63** (DR = 99, and DS = −16.99 kcal/mol) formed H-bonds with the side chains of Asp214 and Thr215, while the sulfone moiety of **63** interacted with the amino group of Thr215 via H-bond. The docked view of compound **94** (DS = 16.47 kcal/mol, DR = 143) showed that the thioxoimidazolidinone moeity of **94** interacts with multiple residues including Asp68, Asp214 and His111 through H-bonds. Additionally, two water molecules also stabilize the compound by H-bonding. The binding mode of compound **112** showed that the triazolopyridine ring of the compound mediated ionic interactions with Asp349, while the substituted hydroxyl group of the compound formed H-bonds with Glu276 and Arg212. The compound depicted DS and DR of −16.27 kcal/mol and 167, respectively. The docked view of **135** showed that one of the nitrile group formed H-bond with His348, while amino group and piperidine nitrogen of the compound mediated H-bonds Asp68 and Glu276, respectively. Moreover, the side chains of Asp68 and Glu276 also offered ionic interaction to the compound. The compound exhibited DS = −16.02 kcal/mol and DR =194. The binding mode of compound **140** (DS = −15.95 kcal/mol, DR = 200) depicted that the hydroxytetrahydropyrimidinone ring of **140** binds with Asp214, Asp349, Arg212 and His348 through H-bonds. 

**Figure 5:**
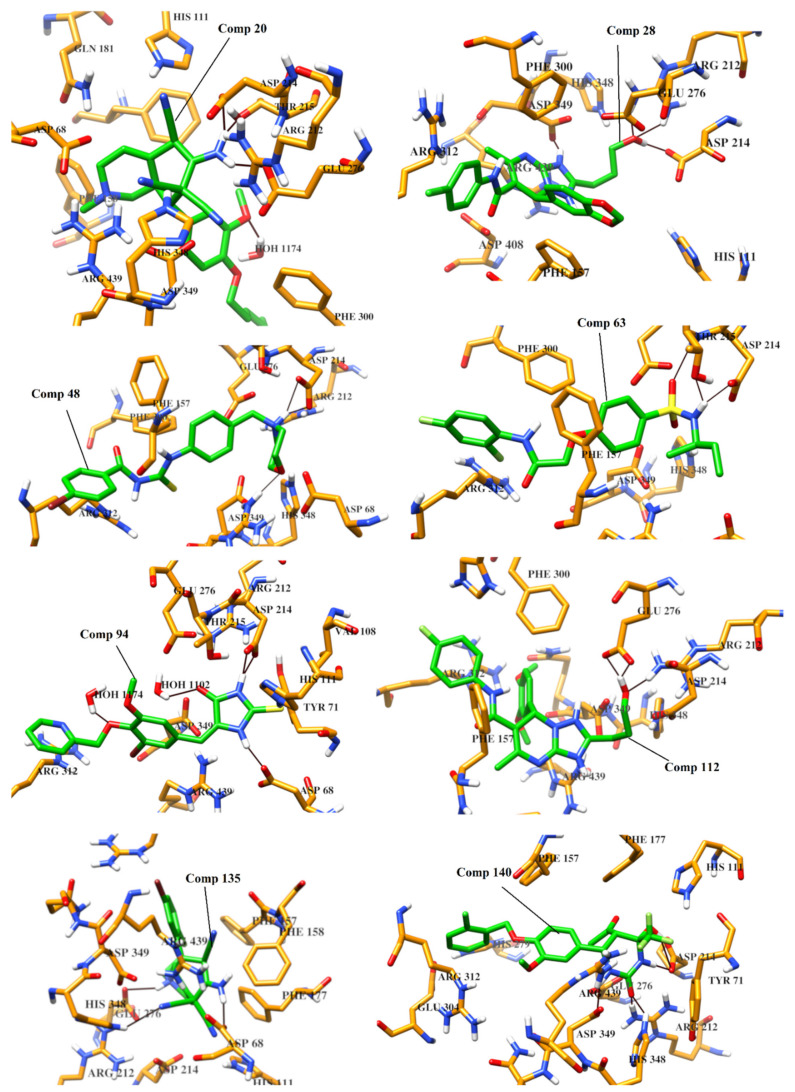
The binding modes of compounds **20, 28, 48, 63, 94, 112, 135** and **140** are depicted in the active site of α-glucosidase. Ligands are shown in green stick model, H-bonds are displayed in black lines, the active site residues are presented in orange stick model.

The binding interactions reflect that these compounds possess strong binding potential with α-glucosidase. The chemical structures of the selected hits, and their binding interactions with the active site residues are shown in Table 2 and docking results are tabulated in Table S2. The 3D-structures of selected hits are given in Table S3. The binding modes of the selected hits are depicted in Figure 5. Therefore, by using virtual screening, we selected most appropriate binders of α-Glucosidase enzyme. Thus, this study will be useful in the identification of novel and more potent anti-diabetic compounds. 

### 2.6. Prediction of α-Glucosidase Inhibitory Activities of Compounds (**20**, **28**, **48**, **63**, **94**, **112**, **135** and **140**) by QSAR Model

The ADMET profile, and predicted binding interactions of compounds 1-142, led us to identify eight new compounds as good inhibitors of α-glucosidase, therefore their α-glucosidase inhibitory activities were predicted by QSAR model. Prior to the prediction, the model was validated by a set of thirty-two compounds (regarded as Test set 2). The compounds in Test set 2 were randomly selected from literature with α-glucosidase inhibitory activities. The developed QSAR model predicted their activities with r^2^ value of 0.62, indicating that the QSAR model can accurately predict the α-glucosidase inhibitory potential of compounds. The results are shown in Table S4 and Figure S4. 

The predicted activities (pIC_50_ values) of compounds **20, 28, 48, 63, 94, 112, 135** and **140** are 5.57, 6.79, 4.04, 4.53, 4.71, 5.85, 5.39, and 4.72, respectively. It reflects that these compounds will serve as better inhibitors when tested in vitro. 

## 3. Methods and Materials

### 3.1. Selection of Data Set for QSAR Analysis

A set of thirty-eight structurally diverse compounds (**αGIs**) with α-glucosidase inhibitory activities was retrieved from literature [42,43,44,45]. **αGIs** covers a good range of inhibitory activities. **αGI1**- **αGI16**, **αGI17**- **αGI33**, and **αGI34**- **αGI38**, are reported by Wang et al. 2017, Taha et al. 2008, and Alhassan et al. 2018, respectively [42,43,44,45]. Based on the diversity of structure and the activity, the data was segregated into training and test sets of 32 and 6 compounds, respectively. The training set was used to develop QSAR model whereas test set was used for the validation of the generated model. The range of biological activities and chemical diversity of compounds was evenly spanned in both sets. The IC_50_ values were converted into negative logarithmic scale (pIC_50_= −logIC_50_) which cover an interval of 3 log units (Table 1). The 3D-cordinates of compounds were built by MOE [46]. Partial charges were applied on compounds and their structures were minimized by MMFF94 force field until gradient was reached to 0.1 kcal/mol/Å^2^ [46]. 

#### The Generation and Validation of QSAR Model

QSAR modeling was performed on MOE. Initially 192 2D-descriptors were calculated for each compound of training set. The best descriptors were selected by using MOE “QuaSAR-Contingency” applications. Descriptors with contingency coefficient above 0.6 and Cramer’s, uncertainty, and correlation coefficients above 0.2 were selected for QSAR which proposed nine 2D-descriptors including apol (Sum of the atomic polarizabilities), bpol (Sum of the absolute value of the difference between atomic polarizabilities of all bonded atoms in the molecule), a_acc (Number of hydrogen bond acceptor atoms), a_heavy (Number of heavy atoms), logP(*o*/*w*) (Log of the octanol/water partition coefficient), logS (Log of the aqueous solubility (mol/L)), TPSA (topological polar surface area (Å2) c), Weight (molecular weight) and wienerPol (Wiener polarity number) as “best” for our dataset. These descriptors are widely used to predict biological activity and ADMET properties of dataset [46]. QSAR model was built by selecting the inhibitory activities of compounds (as descriptor variable) and calculated descriptors as model fields. Regression analysis was performed on the training set, and r^2^ and RMSE values of the fit were obtained. The generated QSAR model was validated by cross validation by leave-one-out method. QSAR fit was used to validate the model by cross validation method. The predictive activities and the residual values of each compound of training set was assessed. Residual value, Z-score and predicted values were calculated for validation and cross-validation of model. The results were interpreted by drawing correlation plot between predicted values (actual activity) on x-axis versus predicted IC_50_ values on y-axis. The developed model was used to predict the biological activities of test set and test set 2 compounds in terms of correlation coefficient (r^2^) between the experimental and predicted activities of test set. The developed QSAR model was used for the prediction of biological activities of selected Hits.

### 3.2. Filtration of ZINC Database for Virtual Screening

A dataset from ZINC database [47] was selected for virtual screening against α-glucosidase. Compounds were searched by using filter parameters calculated by the physicochemical properties of substrate molecule (molecular weight = 150–500, range of xlogP= −4 to 5, number of rotatable bonds = 0–8, maximum topological surface area = 150, number of hydrogen bond donor and acceptor = 0–10, polar desolvation = −400 to 1 kcal/mol, apolar desolvation = −100 to 40 kcal/mol and net charge = −4 to 5). 6609 compounds were matched with the filter parameters that were selected and imported into MOE database, their protonation states were set according to neutral pH, partial charges were applied, and energy was minimized (as described above). 

### 3.3. Docking Based Screening

The crystallographic structure of *Saccharomyces cerevisiae* α-glucosidase is unavailable. Thus, α-glucosidase model was generated in our previous studies [17,18,19]. The homology model was used in the screening of 6609 compounds (collected from ZINC database) and 38 known inhibitors by MOE docking suit [46]. The protonation state of protein model was assigned according to neutral pH, partial charges were applied, and 3D-coordinates of the model was minimized by MMFF94x force field by retaining all heavy atoms fixed until RMSD gradient was reached to 0.1 kcal/mol/Å^2^. Triangle matcher docking algorithm was used with London dG scoring function and GBVI/WSA rescoring method. Force-field based scoring function was applied for post-docking refinement. Later, protein-ligand interaction fingerprint (PLIF) of MOE was used to calculate the binding interactions of compounds within the active site of α-glucosidase. PLIF calculates hydrogen bonding, ionic and hydrophobic interactions [46]. 

### 3.4. Pharmacokinetic (ADMET) Analysis

Pharmacokinetic properties of compounds were calculated by SwissADME (http://www.swissadme.ch/ (accessed on 15 December 2020)) and admetSAR servers (http://lmmd.ecust.edu.cn/ (accessed on 15 December 2020)). These servers are widely used to calculate the absorption, distribution, metabolism, excretion and toxicity (ADMET) of compounds. They predict the drug likeness, lead likeness and synthetic feasibility of compounds. These servers predict the ADMET/drug likeness/lead likeness/pharmacokinetics of compounds via different similarity searching methods or ADMET-QSAR models which are developed by the structures of several drugs or drug like compounds (curated from literature). For ADMET calculation, SMILE format of each compound was uploaded on servers and the obtained results were saved in tabular form. 

## 4. Conclusions

α-Glucosidase inhibitors are class of oral medications which have a promising role in the management of glycemic control in T2DM. In the present study, computational techniques including QSAR modeling and structure-based virtual screening were used to identify potent inhibitors of α-glucosidase. The developed linear QSAR model displayed high predictability with correlation coefficient value (r^2^ = 0.88). The test set validated the model with significant r^2^ (0.82) values. Additionally, virtual screening of 6609 compounds was performed by molecular docking on *S. cerevisiae* α-glucosidase homology model. The best binders were further screened by ADMET profiling and interaction analysis. Eventually eight compounds were selected with high binding affinity for α-glucosidase. Results indicates that those in-silico identified α-glucosidase inhibitors can block the biological activity of α-glucosidase when tested in vitro.

## Figures and Tables

**Table 1:** The chemical structures, α-Glucosidase inhibitory activities, QSAR-predicted activities, and residual values of [α-Glucosidase known Inhibitors (**αGIs**)] used in QSAR modeling.

αGIs	R1	R2	IC_50_ (µM)	pIC_50_	Predicted	Residual
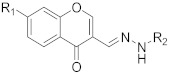						
αGI**1**	H	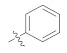	26.7	4.57	4.46	0.11
αGI**2**	H	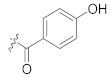	39.8	4.40	4.33	0.07
αGI**3**	H	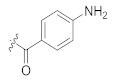	96.9	4.01	4.02	−0.01
αGI**4**	H	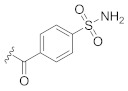	20.1	4.69	4.65	0.04
αGI**5** *	H	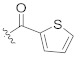	100	4.00	3.83	0.17
αGI**6**	H	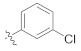	100	4.00	3.88	0.12
αGI**7**	H	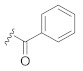	100	4.00	4.07	−0.07
αGI**8**	H	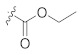	100	4.00	3.89	0.11
αGI**9**	OH	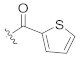	60.8	4.21	4.11	0.10
αGI**10**	H	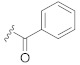	45.7	4.34	4.35	−0.01
αGI**11**	OH	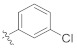	96.7	4.01	4.15	−0.14
αGI**12**	OH	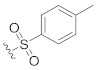	100	4.00	4.09	−0.09
αGI**13**	OH	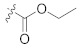	95.4	4.02	4.02	0.00
αGI**14**	OH	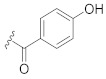	86.3	4.06	4.01	0.05
αGI**15**	H	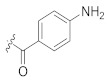	30.8	4.51	4.29	0.22
αGI**16** *	OH	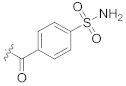	25.2	4.59	4.90	−0.31
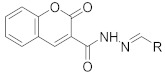						
**α** **G** **Is**	**R**		**IC_50_ (µM)**	**pIC_50_**	**Predicted**	**Residual**
αGI**17**	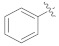		29.14	4.53	4.99	−0.46
αGI**18**	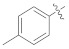		7.58	5.12	4.85	0.27
αGI**19**	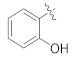		1.1	5.95	5.60	0.35
αGI**20**	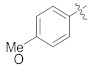		4.26	5.37	4.99	0.38
αGI**21** *	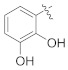		3.15	5.50	5.01	0.49
αGI**22**	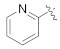		6.1	5.21	5.00	0.21
αGI**23**	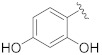		4.58	5.33	5.35	−0.02
αGI**24**	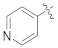		16.1	4.79	4.95	−0.16
αGI**25** *	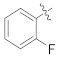		36.46	4.43	4.01	0.42
αGI**26**	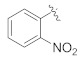		24.14	4.61	4.87	−0.26
αGI**27**	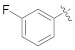		29.14	4.53	4.71	−0.18
αGI**28**	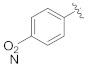		4.58	5.33	5.08	0.25
αGI**29**	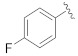		16.1	4.79	4.70	0.09
αGI**30**	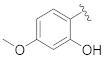		6.46	5.18	5.06	0.12
αGI**31**	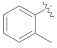		34.14	4.46	4.64	−0.18
αGI**32** *	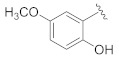		11.14	4.95	5.09	−0.14
αGI**33**	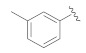		10.58	4.97	4.87	0.10
**α** **G** **Is 34–38**						
αGI**34**	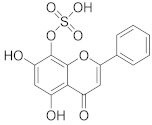		133.57	3.87	3.72	0.15
αGI**35** *	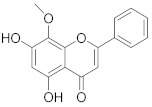		500	3.30	3.40	−0.10
αGI**36**	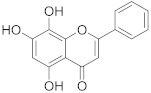		500	3.30	3.68	−0.38
αGI**37**	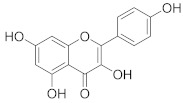		68.46	4.16	3.93	0.23
αGI**38**	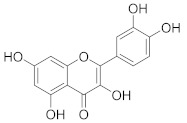		61.86	4.20	4.00	0.20

* Test Set Compounds.

**Table 2:** The Chemical Structures and Binding Interactions of Eight Hits.

Comp	Chemical Formula	Chemical Structure	Interactions with Active Site Residues		
			HB	II	WB
**20**	C25H31N5O2	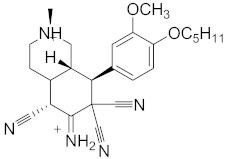	ASP214, GLU276, THR215	ASP68, ASP214, GLU276	HOH1174
**28**	C23H23ClN5O4	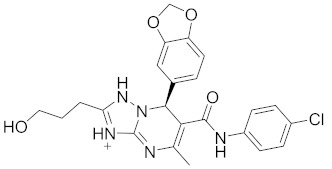	ASP349, ASP214, ARG212	ASP349	none
**48**	C19H21BrN3O2S	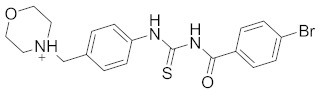	ASP214, ARG439	ASP214	HOH1026
**63**	C18H20F2N2O4S	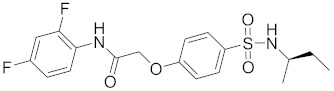	ASP214, THR215	none	none
**94**	C17H14BrN3O3S	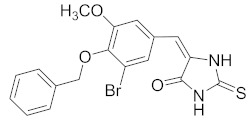	ASP68, ASP214, THR215, HIS111,	none	HOH1102, HOH1174
**112**	C24H27FN5O4	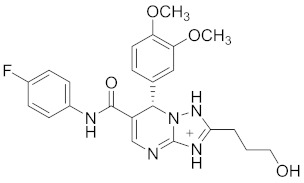	GLU276, ARG212	ASP349	none
**135**	C20H19BrFN5	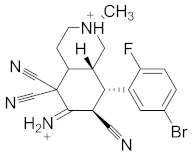	GLU276, ASP68, HIS348	GLU276, ASP68	HOH1026, HOH1228
**140**	C21H20ClF3N2O5	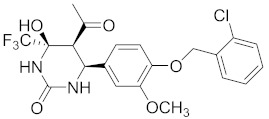	ASP349, ASP214, ARG212, HIS348	none	none

HB= Hydrogen bonds, II = Ionic interactions, WB = water bridging.

## Data Availability

Data is contained within the article or Supplementary Materials.

## Supplementary Materials

The following are available online at https://www.mdpi.com/article/10.3390/ph14050482/s1, Procedure of Homology Modeling and Calculation of Enrichment Factor (EF) and Receiver operating characteristic-curve (ROC-curve), Figure S1: The sequence alignment of S. cerevisiae α-glucosidase model and templates, Figure S2: Receiver Operating Curve (ROC) for α-glucosidase obtained from MOE, Figure S3: correlation of inhibitory activities of known inhibitors with their docking score, Table S1: ADMET Properties of Selected 142 Hits, Table S2: Docking scores, docking ranks and protein-ligand interactions of 142 Hits, Table S3: 3D-structures of selected hits, Table S4: The predicted activities of thirty-two compounds (Test set 2), Figure S4: The correlation plot of Test set 2.
