# Supplementary material for: Rational Design of Novel Inhibitors of α-Glucosidase: An Application of Quantitative Structure Activity Relationship and Structure-Based Virtual Screening
Source: Pharmaceuticals (Basel). 2021 May 19;14(5):482. doi: 10.3390/ph14050482 (PMC8158765; doi:10.3390/ph14050482)
Supplement: Supplementary file 1 [file pharmaceuticals-14-00482-s001.zip › pharmaceuticals-1150475-supplementary.pdf]

Supporting Information

Rational Design of Novel Inhibitors of α-Glucosidase: An Application of Quantitative Structure Activity Relationship and Structure-Based Virtual Screening

Sobia Ahsan Halim [1], Sumaira Jabeen [2], Ajmal Khan [1,*] and Ahmed Al-Harrasi [1,*]

[1] Natural and Medical Sciences Research Center, University of Nizwa, P.O Box 33, 616 Birkat Al Mauz, Nizwa, Oman; sobia_halim@unizwa.edu.om
[2] Department of Chemistry, Government Post-Graduate College for Women, Shadbagh, Lahore 54950, Pakistan; sumairabhatti23@gmail.com
* Correspondence: ajmalchemist@yahoo.com (A.K.); aharrasi@unizwa.edu.om (A.A.-H.); Tel.: +96825446328 (A.A.-H.)

Contents:

- **Procedure of Homology Modeling**
- **Figure S1. The sequence alignment of S. cerevisiae α-glucosidase model and templates**
- **Calculation of Enrichment Factor (EF) and Receiver operating characteristic-curve (ROC-curve)**
- **Figure S2. Receiver Operating Curve (ROC) for α-glucosidase obtained from MOE which showed AUC value of 0.93.**
- **Figure S3. The correlation between Actual activities (pIC50) of known inhibitors (compounds used in QSAR modeling) with their docking scores**
- **Table S1. ADMET Properties of Selected 142 Hits**
- **Table S2. QSAR Predicted pIC_{50} values, docking scores, docking ranks and protein-ligand interactions of 142 Hits**
- **Table S3. 3D-structures of selected hits**
- **Table S4. The predicted activities of thirty-two compounds (Test set 2)**
- **Figure S4. The correlation between Actual activities (pIC50) and Predicted activities ($PRED) of Test set 2 compounds**

Procedure of Homology Modeling

The homology model of *Saccharomyces cerevisiae* was generated by SwissModel server (swissmodel.expasy.org). FASTA sequence of *S. cerevisiae* α-glucosidase was taken from UniProtKB (AC: P53341, www.uniprot.org). *S. cerevisiae* isomaltase (PDB ID: 3AXH, Resolution 1.30Å, 72.4 % identity with *S. cerevisiae* α-glucosidase) was used as template. PROCHECK Ramachandran plot [https://servicesn.mbi.ucla.edu/PROCHECK/], ERRAT plot [https://servicesn.mbi.ucla.edu/ERRAT/] and Verify3D [https://servicesn.mbi.ucla.edu/Verify3D/] were used for structural and physicochemical assessment of model. UCSF Chimera [https://www.cgl.ucsf.edu/chimera/] was used for the visual analysis. Active site residues, catalytic triad and water molecules involve in protein-ligand bridging were identified by superimposing template structure on the model and sequence alignment. Finally, the model was used in the structure-based screening of 6609 compounds.

```
P53341   MTIS-DHPETEPKWWKEATIYQIYPASFKDSNNDGWGDLKGITSKLQYIKDLGVDAIWVCPFYDSPQQDMGY
3AXH     MTISSAHPETEPKWWKEATFYQIYPASFKDSNDDGWGDMKGIASKLEYIKELGADAIWISPFYDSPQDDMGY
3A47     MTISSAHPETEPKWWKEATFYQIYPASFKDSNDDGWGDMKGIASKLEYIKELGADAIWISPFYDSPQDDMGY
cons     ****  *************:************:*****:***:***:***:**.****:.*******:****

P53341   DISNYEKVWPTYGTNEDCFELIDKTHKLGMKFITDLVINHCSTEHEWFKESRSSKTNPKRDWFFWRPPKGYD
3AXH     DIANYEKVWPTYGTNEDCFALIEKTHKLGMKFITDLVINHCSSEHEWFKESRSSKTNPKRDWFFWRPPKGYD
3A47     DIANYEKVWPTYGTNEDCFALIEKTHKLGMKFITDLVINHCSSEHEWFKESRSSKTNPKRDWFFWRPPKGYD
cons     **:**************** **:*******************:*****************************

P53341   AEGKPIPPNNWKSFFGGSAWTFDETTNEFYLRLFASRQVDLNWENEDCRRAIFESAVGFWLDHGVDGFRIDT
3AXH     AEGKPIPPNNWKSYFGGSAWTFDEKTQEFYLRLFCSTQPDLNWENEDCRKAIYESAVGYWLDHGVDGFRIDV
3A47     AEGKPIPPNNWKSYFGGSAWTFDEKTQEFYLRLFCSTQPDLNWENEDCRKAIYESAVGYWLDHGVDGFRIDV
cons     *************:**********.*:*******.* * **********:**:*****:************.

P53341   AGLYSKRPGLPDSPIFDKTSKLQHPNWGSHNGPRIHEYHQELHRFMKNRVKDGREIMTVGEVAHGSDNA--L
3AXH     GSLYSKVVGLPDAPVVDKNSTWQSSDPYTLNGPRIHEFHQEMNQFIRNRVKDGREIMTVGAMQHASDETKRL
3A47     GSLYSKVVGLPDAPVVDKNSTWQSSDPYTLNGPRIHEFHQEMNQFIRNRVKDGREIMTVGEMQHASDETKRL
cons     ..****  ****:*:.**.*. *  .:  :*******:***:::*::************* : *.**::  *

P53341   YTSAARYEVSEVFSFTHVEVGTSPFFRYNIVPFTLKQWKEAIASNFLFINGTDSWATTYIENHDQARSITRF
3AXH     YTSASRHELSELFNFSHTDVGTSPLFRYNLVPFELKDWKIALAELFRYINGTDCWSTIYLENHDQPRSITRF
3A47     YTSASRHELSELFNFSHTDVGTSPLFRYNLVPFELKDWKIALAELFRYINGTDCWSTIYLENHDQPRSITRF
cons     ****:*:*:**:*.*:*.:*****:****:*** **:** *:*. * :*****.*:* *:*****.******

P53341   ADDSPKYRKISGKLLTLLECSLTGTLYVYQGQEIGQINFKEWPIEKYEDVDVKNNYEIIKKSFGKNSKEMKD
3AXH     GDDSPKNRVISGKLLSVLLSALTGTLYVYQGQELGQINFKNWPVEKYEDVEIRNNYNAIKEEHGENSEEMKK
3A47     GDDSPKNRVISGKLLSVLLSALTGTLYVYQGQELGQINFKNWPVEKYEDVEIRNNYNAIKEEHGENSEEMKK
cons     .***** * ******::* .:************:******:**:******:::***: **:..*:**:***.

P53341   FFKGIALLSRDHSRTPMPWTKDKPNAGFTGPDVKPWFLLNESFEQGINVEQESRDDDSVLNFWKRALQARKK
3AXH     FLEAIALISRDHARTPMQWSREEPNAGFSGPSAKPWFYLNDSFREGINVEDEIKDPNSVLNFWKEALKFRKA
3A47     FLEAIALISRDHARTPMQWSREEPNAGFSGPSAKPWFYLNDSFREGINVEDEIKDPNSVLNFWKEALKFRKA
cons     *::.***:****:**** *::::*****:**..**** **:**.:*****:* :* :*******.**: **

P53341   YKELMIYGYDFQFIDLDSDQIFSFTKEYEDKTLFAALNFSGEEIEFSLPREGASLSFILGNYD--DTDVSSR
3AXH     HKDITVYGYDFEFIDLDNKKLFSFTKKYNNKTLFAALNFSSDATDFKIPNDDSSFKLEFGNYPKKEVDASSR
3A47     HKDITVYGYDFEFIDLDNKKLFSFTKKYNNKTLFAALNFSSDATDFKIPNDDSSFKLEFGNYPKKEVDASSR
cons     :*:: :*****:*****..::*****:*::**********.:  :*.:*.:.:*:.: :***   :.*.***

P53341   VLKPWEGRIYLVK
3AXH     TLKPWEGRIYISE
3A47     TLKPWEGRIYISE
cons     .*********: :
```

Figure S1. The sequence alignment of S. cerevisiae α-glucosidase model (P53341) and templates (3AXH and 3A47). The sequence alignment was generated by TCOFFEE (http://tcoffee.crg.cat/).

Calculation of Enrichment Factor (EF) and Receiver operating characteristic-curve (ROC-curve)

EF was calculated by following equation:

$$EF = \frac{\text{HITS sampled/HITS total}}{\text{N sampled/N total}}$$

%EF was calculated by following equation:

$$\%EF = \frac{\text{Enrichment Factor}}{\text{Ideal Enrichment Factor}} \times 100$$

Success was declared when 50% of know α-glucosidase inhibitors (αGIs) were ranked in the top 1% (top 67 compounds), 5% (top 333 compounds) and 10% (top 665 compounds) of screened library. The optimal threshold for virtual screening accuracy was justified by the following criteria: If in top 1%, in top 5% and in top 10% of screened database, all the 38 αGIs are successfully identified then, the ideal EF would be 100, 20 and 10, respectively.

ROC curves differentiate between two populations (active inhibitors and decoy compounds), therefore it is used to evaluate VS performance. ROC curve describes the trade-off between sensitivity (detection of true positives by model) and specificity (ability of model to avoid false negatives). Enrichment is quantified by calculating area under the curve (AUC). If the value of AUC is ≥ 0.9, the scoring function would be considered as excellent, while the value of AUC ≤ 0.6 depicts least or no enrichment. The AUC value of 0.93 was observed for MOE, indicating that MOE is performing well in virtual screening. The ROC curve of MOE is shown in Figure S2.

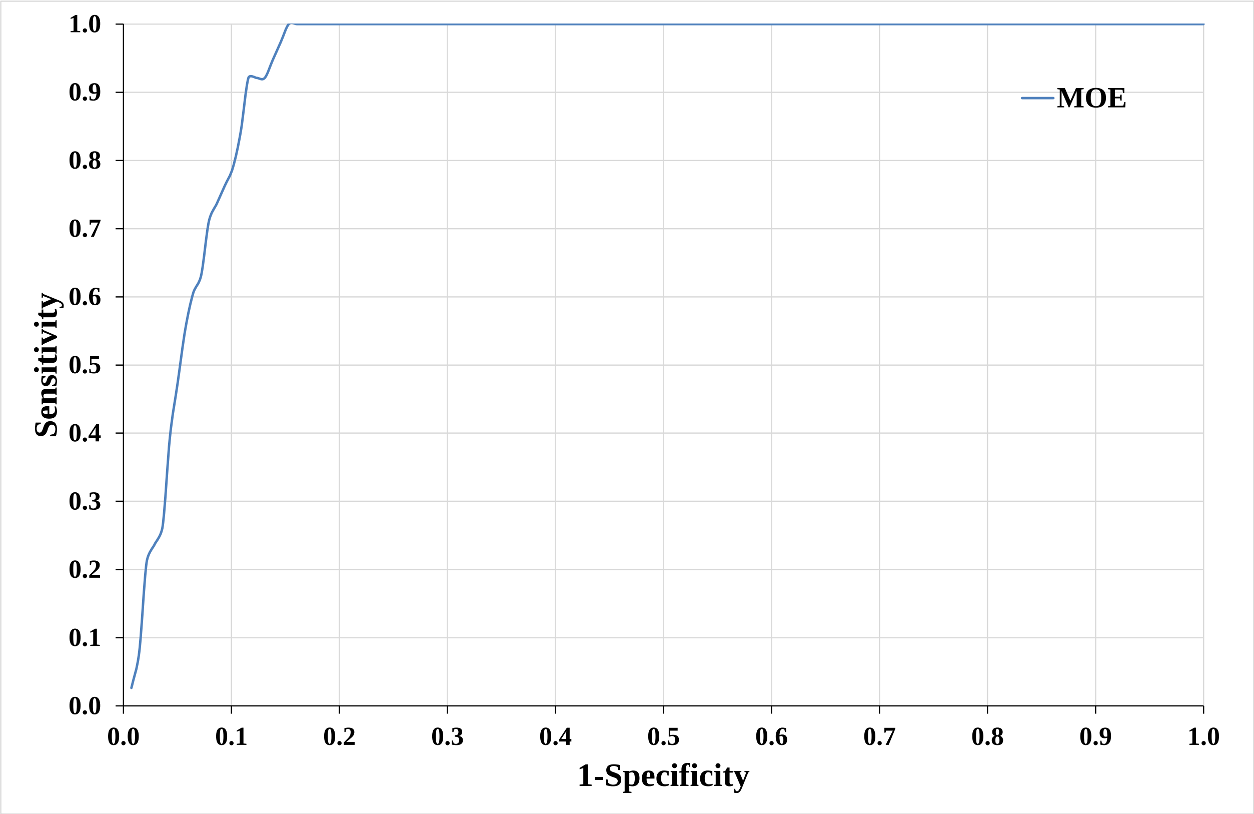

Figure S2. Receiver Operating Curve (ROC) for α-glucosidase obtained from MOE which showed AUC value of 0.94.

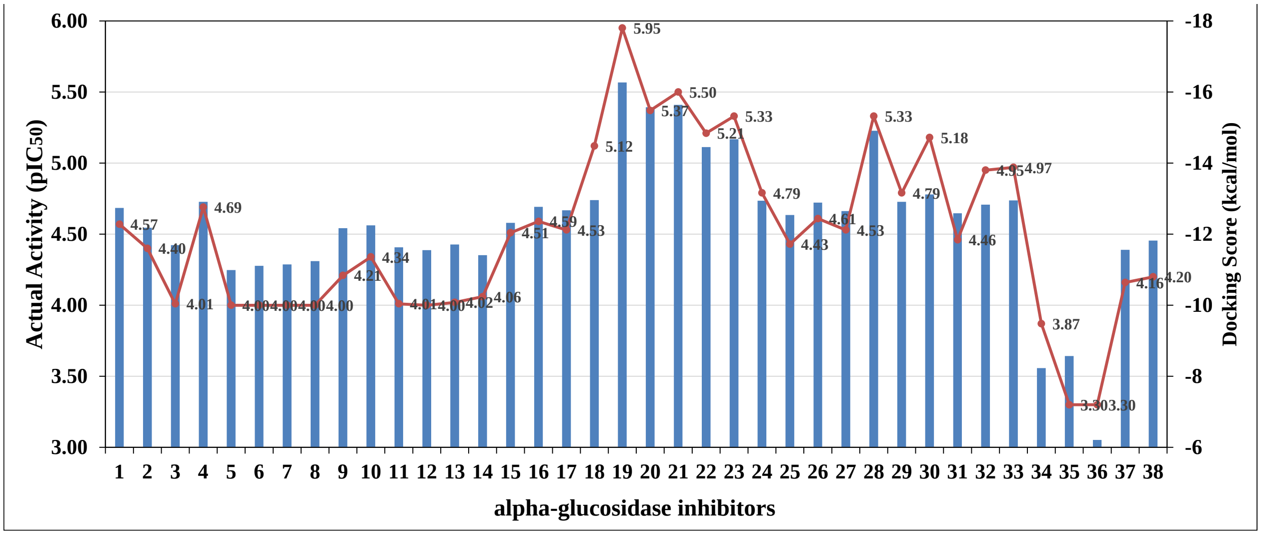

Figure S3. The correlation between Actual activities (pIC_{50}) of known inhibitors (compounds used in QSAR modeling) with their docking scores

Table S1. ADMET Properties of Selected 142 Hits

S#	Docking Rank	ZINC ID	BBBP	HIA	Metabolism	AT	AOT	Carcinogenic	Predicted RAT AT (LD50, mol/kg)	SA
1	2	ZINC00625942	No	High	NS of 2C9, 2D6/Subs: 3A4 NI of 1A2, 2C9, 2D6, 2C19, 3A4	No	III	None	2.2874	4.16
2	3	ZINC08437173	No	High	NS of 2C9, 2D6/Subs: 3A4 NI of 1A2, 2C9, 2D6, 2C19, 3A4	No	III	None	2.5622	3.71
3	5	ZINC08897840	No	High	NS of 2C9, 2D6/Subs: 3A4 NI of 1A2, 2C9, 2D6, 2C19/Inh: 3A4	No	III	None	2.8031	4.75
4	9	ZINC17160090	No	High	NS of 2C9, 2D6/Subs: 3A4 NI of 1A2, 2D6 /Inh : 2C9, 2C19, 3A4	No	III	None	2.4068	3.91
5	14	ZINC08897840	No	High	NS of 2C9, 2D6/Subs: 3A4 NI of 1A2, 2D6, 2C19/Inh: 2C9, 2C19, 3A4	No	III	None	2.6357	4.15
6	15	ZINC08442042	No	High	NS of 2C9, 2D6/Subs: 3A4 NI of 1A2, 2C9, 2D6, 2C19/Inh: 3A4	No	III	None	2.6660	4.95
7	16	ZINC08411460	No	High	NS of 2C9, 2D6/Subs: 3A4 NI of 1A2, 2C9, 2D6, 2C19/Inh: 3A4	No	III	None	2.7487	4.14
8	17	ZINC09357898	No	High	NS of 2C9, 2D6/Subs: 3A4 NI of 1A2, 2D6, 2C19, 3A4/Inh: 2C9	No	III	None	2.5019	3.96
9	18	ZINC08438644	No	High	NS of 2C9, 2D6/Subs: 3A4 NI of 1A2, 2C9, 2D6, 2C19, 3A4	No	III	None	2.5939	3.79
10	19	ZINC08438658	No	High	NS of 2C9, 2D6/Subs: 3A4 NI of 1A2, 2C9, 2D6, 2C19, 3A4	No	III	None	2.5294	3.92
11	21	ZINC17160093	No	High	NS of 2C9, 2D6/Subs: 3A4 NI of 1A2, 2D6, /Inh: 2C9, 2C19, 3A4	No	III	None	2.4068	3.91
12	23	ZINC00625908	No	High	NS of 2C9, 2D6/Subs: 3A4 NI of 1A2, 2C9, 2D6, 2C19, 3A4	No	III	None	2.3654	3.95
13	24	ZINC08429954	No	High	NS of 2C9, 2D6/Subs: 3A4 NI of 1A2, 2D6, 3C19, 3A4/Inh: 2C9	No	III	None	2.3458	3.59
14	25	ZINC00711743	No	High	NS of 2C9, 2D6, 3A4/ NI of 2C9, 2D6, 3A4/Inh: 1A2, 2C19	Yes	III	None	2.3628	3.69
15	29	ZINC08441316	No	High	NS of 2C9, 2D6/Subs: 3A4 NI of 1A2, 2C9, 2D6, 2C19, 3A4	No	III	None	2.5747	4.25
16	30	ZINC04473388	No	High	NS of 2C9, 2D6/Subs: 3A4 NI of 1A2, 2C9, 2D6, 2C19/Inh: 3A4	No	III	None	2.8109	4.36
17	31	ZINC08411329	No	High	NS of 2C9, 2D6/Subs: 3A4 NI of 1A2, 2C9, 2D6, 2C19, 3A4	No	III	None	2.7822	4.53
18	32	ZINC08440549	No	High	NS of 2C9, 2D6/Subs: 3A4 NI of 1A2, 2C9, 2D6, 2C19, 3A4	No	III	None	2.5495	3.65
19	33	ZINC08437172	No	High	NS of 2C9, 2D6/Subs: 3A4 NI of 1A2, 2C9, 2D6, 2C19, 3A4	No	III	None	2.5622	3.71
20	34	ZINC08441930	No	High	NS of 2C9, 2D6/Subs: 3A4 NI of 1A2, 2C9, 2D6, 2C19/Inh: 3A4	No	III	None	2.8031	4.75
21	36	ZINC08438652	No	High	NS of 2C9, 2D6/Subs: 3A4 NI of 1A2, 2C9, 2D6, 2C19, 3A4	No	III	None	2.5149	4.26
22	37	ZINC08441928	No	High	NS of 2C9, 2D6/Subs: 3A4 NI of 1A2, 2C9, 2D6, 2C19/Inh: 3A4	No	III	None	2.8031	4.75
23	38	ZINC08432474	No	High	NS of 2C9, 2D6, 3A4/NI of 1A2, 2D6, 3A4/Inh: 2C9, 2C19	No	III	None	2.4481	3.69
24	42	ZINC17160093	No	High	NS of 2C9, 2D6/Subs: 3A4 NI of 1A2, 2D6/Inh: 2C9, 2C19, 3A4	No	III	None	2.4068	4.01
25	43	ZINC05313283	No	High	NS of 2C9, 2D6/Subs: 3A4 NI of 1A2, 2C9, 2D6, 2C19, 3A4	No	III	None	2.5470	3.23

26	44	ZINC08438660	No	High	NS of 2C9, 2D6/Subs: 3A4 NI of 1A2, 2C9, 2D6, 2C19, 3A4	No	III	None	2.5294	3.92
27	45	ZINC08438544	No	High	NS of 2C9, 2D6/Subs: 3A4 NI of 1A2, 2C9, 2D6, 2C19, 3A4	No	III	None	2.6553	3.70
28	46	ZINC08437141	No	High	NS of 2C9, 2D6/Subs: 3A4 NI of 1A2, 2C9, 2D6, 2C19/Inh: 3A4	No	III	None	2.6140	4.55
29	48	ZINC08430547	No	High	NS of 2C9, 2D6/Subs: 3A4 NI of 1A2, 2C9, 2D6, 2C19/Inh: 3A4	No	III	None	2.5604	3.63
30	51	ZINC00625938	No	High	NS of 2C9, 2D6/Subs: 3A4 NI of 1A2, 2C9, 2D6, 2C19, 3A4	No	III	None	2.2874	4.16
31	53	ZINC00625757	No	High	NS of 2C9, 2D6/Subs: 3A4 NI of 1A2, 2D6, 2C19, 3A4 /Inh: 2C9	No	III	None	2.3226	4.04
32	54	ZINC00146770	No	High	NS of 2C9, 2D6, 3A4 /NI of 2C9, 2D6, 3A4 /Inh: 1A2, 2C19	No	III	None	2.2305	3.45
33	57	ZINC08438548	No	High	NS of 2C9, 2D6/Subs: 3A4 NI of 1A2, 2C9, 2D6, 2C19, 3A4	No	III	None	2.5866	4.12
34	58	ZINC00626525	No	High	NS of 2C9, 2D6 Subs: 3A4 NI of 1A2, 2D6, 2C19, Inh:2C9, 3A4	No	III	None	2.7568	3.81
35	59	ZINC08431434	No	High	NS of 2C9, 2D6/Subs: 3A4 /Inh: 1A2, 2C9, 2D6, 2C19, 3A4	No	III	None	2.6779	3.12
36	60	ZINC08411476	No	High	NS of 2C9, 2D6/Subs: 3A4 NI of 2D6, 1A2, 2C9, 2C19/Inh: 3A4	No	III	None	2.7487	4.14
34	61	ZINC05313297	No	High	NS of 2C9, 2D6/Subs: 3A4 NI of 2D6, 1A2, 2C9, 2C19, 3A4	No	III	None	2.2890	3.99
38	62	ZINC08437656	No	High	NS of 2C9, 2D6/Subs: 3A4 NI of 2D6, 1A2, 2C9, 3A4/Inh: 2C19	No	III	None	2.7198	3.17
39	64	ZINC09070021	No	High	NS of 2C9, 2D6/Subs: 3A4 NI of 1A2, 2C9, 2D6, 2C19, 3A4	No	III	None	2.5569	4.41
40	65	ZINC02159737	No	High	NS of 2C9, 2D6/Subs: 3A4 NI of 1A2, 2C9, 2D6, 2C19, 3A4	No	III	None	2.5576	3.66
41	67	ZINC08438546	No	High	NS of 2C9, 2D6/Subs: 3A4 NI of 1A2, 2C9, 2D6, 2C19, 3A4	No	III	None	2.6553	3.70
42	70	ZINC06144562	No	High	NS of 2C9, 2D6/Subs: 3A4 NI of 1A2, 2C9, 2D6, 2C19/Inh: 3A4	No	III	None	2.6033	4.55
43	71	ZINC01413496	No	High	NS of 2C9, 2D6/Subs: 3A4 NI of 1A2, 2C9, 2D6, 2C19/Inh: 3A4	No	III	None	2.6476	3.78
44	72	ZINC00703045	No	High	NS of 2C9, 2D6/Subs: 3A4 NI of 1A2, 2D6, 2C19, 3A4/Inh: 2C9	No	III	None	2.5338	4.41
45	74	ZINC06904335	No	High	NS of 2C9, 2D6, 3A4 NI of 2D6, 3A4/Inh:1A2, 2C9, 2C19	No	III	None	2.4791	3.16
46	75	ZINC08437908	Yes	High	NS of 2C9, 2D6, 3A4 NI of 2C9 Inh:1A2, 2D6, 2C19, 3A4	No	III	None	2.4771	2.81
47	76	ZINC00702446	No	High	NS of 2C9, 2D6/Subs: 3A4 NI of 1A2, 2D6, /Inh: 2C9, 2C19, 3A4	No	III	None	2.5607	3.86
48	77	ZINC08437778	No	High	NS of 2C9, 2D6, 3A4 NI of 1A2, 2D6, 3A4/ Inh: 2C9, 2C19	No	III	None	2.5821	2.61
49	78	ZINC08435576	No	High	NS of 2C9, 2D6/Subs: 3A4 NI of 1A2, 2C9, 2D6, 2C19/Inh: 3A4	No	III	None	2.4160	3.95
50	79	ZINC08438656	No	High	NS of 2C9, 2D6/Subs: 3A4 NI of 1A2, 2C9, 2D6, 2C19, 3A4	No	III	None	2.7125	3.89
51	82	ZINC00626165	No	High	NS of 2C9, 2D6/Subs: 3A4 NI of 1A2, 2D6, 2C19, 3A4/Inh: 2C9	No	III	None	2.2696	3.90
52	83	ZINC00702307	No	High	NS of 2C9, 2D6/Subs: 3A4 NI of 1A2, 2D6, 3A4/Inh: 2C9, 2C19	No	III	None	2.6782	3.67

53	84	ZINC08438550	No	High	NS of 2C9, 2D6/Subs: 3A4 NI of 1A2, 2C9, 2D6, 2C19, 3A4	No	III	None	2.5866	4.12
54	86	ZINC08432903	No	High	NS of 2C9, 2D6/Subs: 3A4 NI of 1A2, 2C9, 2D6, 2C19/Inh: 3A4	No	III	None	2.3286	3.30
55	87	ZINC00703050	No	High	NS of 2C9, 2D6/Subs: 3A4 NI of 1A2, 2C9, 2D6, 2C19, 3A4	No	III	None	2.5822	4.13
56	89	ZINC08440218	No	High	NS of 2C9, 2D6/Subs: 3A4 NI of 1A2, 2D6 Inh: 2C9, 2C19, 3A4	No	III	None	2.5607	3.86
57	90	ZINC08432475	No	High	NS of 2C9, 2D6, 3A4 NI of 1A2, 2D6, 3A4/ Inh: 2C9, 2C19	No	III	None	2.4481	3.69
58	91	ZINC08442014	No	High	NS of 2C9, 2D6/Subs: 3A4 NI of 1A2, 2C9, 2D6, 2C19/Inh: 3A4	No	III	None	2.7165	5.03
59	92	ZINC00625761	No	High	NS of 2C9, 2D6/Subs: 3A4 NI of 1A2, 2D6, 2C19, 3A4/Inh: 2C9	No	III	None	2.3226	4.04
60	95	ZINC17160093	No	High	NS of 2C9, 2D6/Subs: 3A4 NI of 1A2, 2D6 Inh: 2C9, 2C19, 3A4	No	III	None	2.1764	3.85
61	96	ZINC08435575	No	High	NS of 2C9, 2D6/Subs: 3A4 NI of 1A2, 2C9, 2D6, 2C19/Inh: 3A4	No	III	None	2.2590	3.84
62	97	ZINC00857151	No	High	NS of 2C9, 2D6/Subs: 3A4 NI of 1A2, 2C9, 2D6, 3A4/Inh: 2C19	No	III	None	2.3238	3.12
63	99	ZINC00983605	No	High	NS of 2C9, 2D6/Subs: 3A4 NI of 1A2, 2D6, 3A4/Inh: 2C9, 2C19	No	III	None	2.4080	3.24
64	100	ZINC15952849	No	High	NS of 2C9, 2D6/Subs: 3A4 NI of 1A2, 2C9, 2D6, 2C19, 3A4	No	III	None	2.5438	4.52
65	101	ZINC08437196	No	High	NS of 2C9, 2D6/Subs: 3A4 NI of 1A2, 2C9, 2D6, 2C19/Inh: 3A4	No	III	None	2.5712	4.84
66	102	ZINC00703108	No	High	NS of 2C9, 2D6/Subs: 3A4 NI of 1A2, 2D6, 2C19, 3A4/Inh: 2C9	No	III	None	2.4043	4.29
67	104	ZINC08435580	No	High	NS of 2C9, 2D6/Subs: 3A4 NI of 1A2, 2C9, 2D6, 2C19, 3A4	No	III	None	2.5065	3.91
68	105	ZINC02159741	No	High	NS of 2C9, 2D6/Subs: 3A4 NI of 1A2, 2C9, 2D6, 3A4/Inh: 2C19	No	III	None	2.6893	3.44
69	106	ZINC08417536	No	High	NS of 2C9, 2D6, 3A4 NI of 1A2, 2C9, 2D6, 2C19, 3A4	No	III	None	2.1645	2.47
70	107	ZINC09008533	No	High	NS of 2C9, 2D6/Subs: 3A4 NI of 1A2, 2C9, 2D6, 2C19, 3A4	No	III	None	2.3979	3.52
71	108	ZINC08441623	No	High	NS of 2C9, 2D6/Subs: 3A4 NI of 1A2, 2C9, 2D6, 2C19, 3A4	No	III	None	2.6423	4.57
72	109	ZINC08441892	No	High	NS of 2C9, 2D6/Subs: 3A4 NI of 1A2, 2C9, 2D6, 2C19/Inh: 3A4	No	III	None	2.7922	4.87
73	111	ZINC08437653	No	High	NS of 2C9, 2D6/Subs: 3A4 NI of 1A2, 2C9, 2D6, 3A4/Inh: 2C19	No	III	None	2.6135	3.48
74	112	ZINC08996455	No	High	NS of 2C9, 2D6/Subs: 3A4 NI of 1A2, 2C9, 2D6, 2C19, 3A4	No	III	None	2.4253	4.23
75	113	ZINC00832890	No	High	NS of 2C9, 2D6/Subs: 3A4 NI of 1A2, 2C9, 2D6, 2C19, 3A4	No	III	None	2.2676	2.78
76	117	ZINC06144561	No	High	NS of 2C9, 2D6/Subs: 3A4 NI of 1A2, 2C9, 2D6, 2C19/Inh: 3A4	No	III	None	2.6033	4.55
77	120	ZINC08435579	No	High	NS of 2C9, 2D6/Subs: 3A4 NI of 1A2, 2C9, 2D6, 2C19, 3A4	No	III	None	2.3668	3.66
78	123	ZINC00625713	No	High	NS of 2C9, 2D6/Subs: 3A4 NI of 1A2, 2C9, 2D6, 2C19, 3A4	No	III	None	2.1747	4.24
79	124	ZINC08441947	No	High	NS of 2C9, 2D6/Subs: 3A4 NI of 1A2, 2C9, 2D6, 2C19/Inh: 3A4	No	III	None	2.8140	4.54

80	125	ZINC08897821	No	High	NS of 2C9, 2D6/Subs: 3A4 NI of 1A2, 2D6, Inh: 2C9, 2C19, 3A4	No	III	None	2.6357	4.04
81	127	ZINC09046651	No	High	NS of 2C9, 2D6/Subs: 3A4 NI of 1A2, 2C9, 2D6, 2C19, 3A4	No	III	None	2.6241	3.83
82	129	ZINC08432386	No	High	NS of 2C9, 2D6/Subs: 3A4 NI of 1A2, 2D6, Inh: 2C9, 2C19, 3A4	No	III	None	2.4239	3.56
83	130	ZINC17163315	No	High	NS of 2C9, 2D6/Subs: 3A4 NI of 1A2, 2D6, 3A4/Inh: 2C9, 2C19	No	III	None	2.6652	3.79
84	131	ZINC00711757	No	High	NS of 2C9, 2D6/Subs: 3A4 NI of 2C9,2D6, 2C19, 3A4/Inh: 1A2	No	III	None	2.3503	3.72
85	132	ZINC08440772	No	High	NS of 2C9, 2D6/Subs: 3A4 NI of 1A2, 2D6, 3A4/Inh: 2C9, 2C19	No	III	None	2.4859	3.71
86	133	ZINC08441928	No	High	NS of 2C9, 2D6/Subs: 3A4 NI of 1A2, 2C9, 2D6, 2C19/Inh: 3A4	No	III	None	2.8227	4.71
87	134	ZINC20032743	No	High	NS of 2C9, 2D6/Subs: 3A4 NI of 1A2, 2C9, 2D6, 2C19, 3A4	No	III	None	2.6780	4.72
88	135	ZINC08414138	No	High	NS of 2C9, 2D6, 3A4/ NI of 1A2, 2C9, 2D6, 2C19, 3A4	No	III	None	2.2296	4.27
89	137	ZINC08897821	No	High	NS of 2C9, 2D6/Subs: 3A4 NI of 1A2, 2D6, Inh: 2C9, 2C19, 3A4	No	III	None	2.6357	4.15
90	138	ZINC01413485	No	High	NS of 2C9, 2D6/Subs: 3A4 NI of 1A2, 2C9, 2D6, 2C19, 3A4	No	III	None	2.6513	3.70
91	139	ZINC08411504	No	High	NS of 2C9, 2D6/Subs: 3A4 NI of 1A2, 2C9 2D6, 3A4/Inh: 2C19	No	III	None	2.7955	4.44
92	140	ZINC05286115	No	High	NS of 2C9, 2D6, 3A4 NI of 2C9 2D6, 2C19, 3A4 Inh: 1A2	No	III	None	2.2074	3.08
93	141	ZINC08433219	No	High	NS of 2C9, 2D6/Subs: 3A4 NI of 1A2, 2C9, 2D6, 2C19, 3A4	No	III	None	2.5033	4.17
94	143	ZINC00683816	No	High	NS of 2C9, 2D6/Subs: 3A4 NI of 2D6 /Inh: 1A2, 2C9, 2C19, 3A4	No	III	None	2.4657	3.11
95	144	ZINC00292755	Yes	High	NS of 2C9, 2D6, 3A4 NI of 2C9 Inh: 1A2, 2D6, 2C19, 3A4	No	III	None	2.5695	1.99
96	145	ZINC01414766	No	High	NS of 2C9, 2D6/Subs: 3A4 NI of 1A2, 2C9, 2D6, 2C19/Inh: 3A4	No	III	None	2.6588	3.64
97	146	ZINC06444803	No	High	NS of 2C9, 2D6/Subs: 3A4 NI of 1A2, 2C9, 2D6, 2C19, 3A4	No	III	None	2.2865	4.10
98	147	ZINC08427102	No	High	NS of 2C9, 2D6/Subs: 3A4 NI of 1A2, 2C9, 2D6, 2C19/Inh: 3A4	No	III	None	2.8059	4.40
99	148	ZINC17160090	No	High	NS of 2C9, 2D6/Subs: 3A4 NI of 1A2, 2D6, Inh: 2C9, 2C19, 3A4	No	III	None	2.4819	3.88
100	149	ZINC08438781	No	High	NS of 2C9, 2D6/Subs: 3A4 NI of 1A2, 2C9, 2D6, 2C19, 3A4	No	III	None	2.6589	3.78
101	150	ZINC08439872	No	High	NS of 2C9, 2D6/Subs: 3A4 NI of 1A2, 2C9, 2D6, 2C19, 3A4	No	III	None	2.4875	3.78
102	152	ZINC19989886	No	High	NS of 2C9, 2D6/Subs: 3A4 NI of 1A2, 2C9, 2D6, 2C19, 3A4	No	III	None	2.6528	3.43
103	153	ZINC08441926	No	High	NS of 2C9, 2D6/Subs: 3A4 NI of 1A2, 2C9, 2D6, 2C19/Inh: 3A4	No	III	None	2.8031	4.75
104	154	ZINC08432384	No	High	NS of 2C9, 2D6, 3A4 NI of 2C9 Inh:1A2, 2D6, 2C19, 3A4	No	III	None	2.4985	3.26
105	157	ZINC08414555	No	High	NS of 2C9, 2D6/Subs: 3A4 NI of 1A2, 2D6, Inh: 2C9, 2C19, 3A4	No	III	None	2.3962	3.85
106	159	ZINC00646487	No	High	NS of 2C9, 2D6/Subs: 3A4 NI of 2D6, 2C19 Inh: 1A2, 2C9, 3A4	No	III	None	2.5795	4.59

107	160	ZINC09046768	No	High	NS of 2C9, 2D6/Subs: 3A4 NI of 1A2, 2C9, 2D6, 2C19, 3A4	No	III	None	2.6584	4.32
108	162	ZINC00692735	No	High	NS of 2C9, 2D6/Subs: 3A4 NI of 1A2, 2C9, 2D6, 3A4/Inh: 2C19	No	III	None	2.3881	3.03
109	163	ZINC02162053	No	High	NS of 2C9, 2D6/Subs: 3A4 NI of 2D6, 3A4 Inh:1A2, 2C9, 2C19	No	III	None	2.6042	3.38
110	165	ZINC08441462	No	High	NS of 2C9, 2D6/Subs: 3A4 NI of 1A2, 2C9, 2D6, 2C19, 3A4	No	III	None	2.4768	4.70
111	166	ZINC08437911	Yes	High	NS of 2C9, 2D6, 3A4 /NI of 2C9, Inh: 1A2, 2D6, 2C19,3A4	No	III	None	2.4771	2.79
112	167	ZINC01795564	No	High	NS of 2C9, 2D6/Subs: 3A4 NI of 1A2, 2C9, 2D6, 2C19/Inh: 3A4	No	III	None	2.5882	4.72
113	168	ZINC01413497	No	High	NS of 2C9, 2D6/Subs: 3A4 NI of 1A2, 2C9, 2D6, 2C19/Inh: 3A4	No	III	None	2.6476	3.78
114	169	ZINC08429897	No	High	NS of 2C9, 2D6/Subs: 3A4 NI of 2D6, 3A4 Inh:1A2, 2C9, 2C19	No	III	None	2.6407	3.84
115	171	ZINC08437218	No	High	NS of 2C9, 2D6/Subs: 3A4 NI of 1A2, 2C9, 2D6, 2C19/Inh: 3A4	No	III	None	2.6421	4.92
116	172	ZINC00665548	No	High	NS of 2C9, 2D6/Subs: 3A4 NI of 1A2, 2D6, Inh: 2C9, 2C19, 3A4	No	III	None	2.5292	3.01
117	173	ZINC08437199	No	High	NS of 2C9, 2D6/Subs: 3A4 NI of 1A2, 2D6, Inh: 2C9, 2C19, 3A4	No	III	None	2.5439	5.16
118	174	ZINC00702987	No	High	NS of 2C9, 2D6/Subs: 3A4 NI of 1A2, 2C9, 2D6, 2C19, 3A4	No	III	None	2.8256	4.34
119	175	ZINC00630492	No	High	NS of 2C9, 2D6/Subs: 3A4 NI of 2D6/Inh: 1A2,2C9, 2C19, 3A4	No	III	None	2.2816	4.02
120	177	ZINC08441601	No	High	NS of 2C9, 2D6/Subs: 3A4 NI of 2D6 /Inh: 1A2, 2C9, 2C19, 3A4	No	III	None	2.6660	5.09
121	178	ZINC02067899	Yes	High	NS of 2C9, 2D6, 3A4 /NI of 2D6 3A4 / Inh: 1A2, 2C9, 2C19	No	III	None	2.5076	2.97
122	179	ZINC08430546	No	High	NS of 2C9, 2D6, 3A4 NI of 1A2, 2C9, 2D6, 2C19 Inh: 3A4	No	III	None	2.5333	3.65
123	180	ZINC08431354	No	High	NS of 2C9, 2D6, 3A4 NI of 2C9, 2D6, 2C19, 3A4 Inh: 1A2	No	III	None	2.4481	3.17
124	181	ZINC00984875	No	High	NS of 2C9, 2D6/Subs: 3A4 NI of 1A2, 2C9, 2D6, 2C19/Inh: 3A4	No	III	None	2.2982	3.86
125	182	ZINC02479986	No	High	NS of 2C9, 2D6, 3A4 NI of 1A2, 2C9, 2D6, 2C19, 3A4	No	III	None	2.5472	3.62
126	183	ZINC08441602	No	High	NS of 2C9, 2D6/Subs: 3A4 NI of 1A2, 2D6, 3A4 /Inh: 2C9, 2C19	No	III	None	2.4508	3.77
127	184	ZINC00702427	No	High	NS of 2C9, 2D6/Subs: 3A4 NI of 1A2, 2D6, 3A4 /Inh: 2C9, 2C19	No	III	None	2.9770	3.77
128	185	ZINC08414444	Yes	High	NS of 2C9, 2D6/Subs: 3A4 NI of 1A2, 2C9, 2D6 /Inh: 2C19, 3A4	No	III	None	2.4908	4.13
129	186	ZINC06144543	No	High	NS of 2C9, 2D6/Subs: 3A4 NI of 1A2, 2C9, 2D6, 2C19/Inh: 3A4	No	III	None	2.6320	4.45
130	187	ZINC08411455	No	High	NS of 2C9, 2D6/Subs: 3A4 NI of 1A2, 2C9, 2D6, 2C19, 3A4	No	III	None	2.6989	436
131	188	ZINC08429898	No	High	NS of 2C9, 2D6/Subs: 3A4 NI of 2D6, 3A4 Inh:1A2, 2C9, 2C19	No	III	None	2.6407	3.84
132	190	ZINC01952505	No	High	NS of 2C9, 2D6/Subs: 3A4 NI of 1A2, 2C9, 2D6, 3A4 /Inh: 2C19	No	III	None	2.6063	3.71
133	191	ZINC08433001	No	High	NS of 2C9, 2D6, 3A4 NI of 1A2, 2C9, 2D6, 2C19 Inh: 3A4	No	III	None	2.3507	3.97

134	192	ZINC08425651	No	High	NS of 2C9, 2D6/Subs: 3A4 NI of 1A2, 2C9, 2D6, 2C19, 3A4	No	III	None	2.6947	4.48
135	194	ZINC05919492	No	High	NS of 2C9, 2D6, 3A4 NI of 1A2, 2C9, 2D6, 2C19, 3A4	No	III	None	2.7030	4.37
136	195	ZINC08436971	No	High	NS of 2C9, 2D6/Subs: 3A4 NI of 1A2, 2D6, 2C19, 3A4 /Inh: 2C9	No	III	None	2.6982	3.21
137	196	ZINC08817408	Yes	High	NS of 2C9, 2D6/Subs: 3A4 NI of 1A2, 2C9, 2D6, 2C19, 3A4	No	III	None	2.6672	3.68
138	197	ZINC08411308	No	High	NS of 2C9, 2D6/Subs: 3A4 NI of 1A2, 2C9, 2D6, 3A4 /Inh: 2C19	No	III	None	2.7551	4.75
139	199	ZINC08427099	No	High	NS of 2C9, 2D6/Subs: 3A4 NI of 1A2, 2C9, 2D6, 2C19/Inh: 3A4	No	III	None	2.7109	4.41
140	200	ZINC08437082	No	High	NS of 2C9, 2D6/Subs: 3A4 NI of 1A2, 2C9, 2D6, 2C19, 3A4	No	III	None	2.3994	4.02
141	201	ZINC08437193	No	High	NS of 2C9, 2D6/Subs: 3A4 NI of 1A2, 2C9, 2D6, 2C19/Inh: 3A4	No	III	None	2.5712	4.84
142	202	ZINC08417802	No	High	NS of 2C9, 2D6, 3A4/NI of 1A2, 2C9, 2D6, 2C19, 3A4	No	III	None	2.4833	3.06

BBBP: blood brain barrier permeant, HIA: human intestinal absorption, NS: non-substrates, NI: non-inhibitor, Subs: substrate, Inh: inhibitor, AT: AMES toxicity, AOT: acute oral toxicity, SA: synthetic accessibility. The compounds that show excellent pharmacokinetic profile are highlighted with green color.

Table S2. The docking scores, docking ranks and protein-ligand interactions of 142 Hits

Compounds	QSAR Predicted Activity	Docking Rank	ZINC ID	Docking Score	Protein-ligand Interactions			
					LA	RA	Bond	Distance (Å)
1	6.32	2	ZINC00625942	-21.43	N63	OE1-GLU276	HBD	3.36
					N47	OD1-ASP214	ionic	3.87
					N47	OD2-ASP214	ionic	3.82
					N47	OE1-GLU276	ionic	3.64
					N63	OE1-GLU276	ionic	3.36
					N63	OE2-GLU276	ionic	2.99
2	6.96	3	ZINC08437173	-21.16	N52	OD1-ASP349	HBD	3.28
					O13	O-HOH1174	HBA	2.08
					O51	NE-ARG312	ionic	2.60
					O51	NH2-ARG312	ionic	3.44
					N52	OD1-ASP349	ionic	3.28
					N52	OD2-ASP349	ionic	2.85
3		5	ZINC08897840	-20.34	N50	O-HOH1174	HBA	3.12
					N54	NH2-ARG212	HBA	2.65
					N54	NE2-HIS348	HBA	2.71
					N59	OD1-ASP68	ionic	3.24
					N59	OD2-ASP68	ionic	2.95
					N61	OE1-GLU276	ionic	2.53
					N61	OE2-GLU276	ionic	1.79
4		9	ZINC17160090	-19.57	N42	OD1-ASP214	ionic	3.67
					N42	OE1-GLU276	ionic	3.62
					N42	OE2-GLU276	ionic	3.68
					N42	OD2-ASP349	ionic	3.41
					N47	OD1-ASP214	ionic	2.61
					N47	OD2-ASP214	ionic	3.68
					O51	NE-ARG312	ionic	2.57
					O51	NH1-ARG312	ionic	3.67
					O51	NH2-ARG312	ionic	2.94
5		14	ZINC08897840	-19.16	O30	NE-ARG312	ionic	2.57
					O30	NH2-ARG312	ionic	3.14
					N45	OD2-ASP68	ionic	3.53
					N52	OD1-ASP214	ionic	3.35
					N52	OD2-ASP214	ionic	2.86
6		15	ZINC08442042	-19.10	N42	OD1-ASN241	HBD	3.45
					O31	NE2-HIS348	HBA	3.06
					5-ring	6-ring-PHE157	π-π	3.43
7		16	ZINC08411460	-19.10	N30	O-HOH1056	HBA	2.77
					N42	OD1-ASP349	ionic	2.53
					N42	OD2-ASP349	ionic	2.71
8		17	ZINC09357898	-19.08	N27	OD2-ASP214	ionic	3.26
					N32	OD1-ASP214	ionic	2.52
					N32	OD2-ASP214	ionic	1.44
					O36	NE-ARG439	ionic	3.43
					6-ring	O-HOH1174	H-π	3.46
9		18	ZINC08438644	-19.07	O40	NE-ARG312	ionic	1.89
					O40	NH1-ARG312	ionic	2.97
					O40	NH2-ARG312	ionic	1.89
					O42	NE-ARG312	ionic	3.45
					O42	NH1-ARG312	ionic	3.62
					N56	OD2-ASP349	ionic	3.75
10		19	ZINC08438658	-19.05	O17	NE-ARG312	HBA	3.32
					O17	NE-ARG312	ionic	3.32
					O57	NE-ARG312	ionic	2.29
					O57	NH1-ARG312	ionic	3.34
					O57	NH2-ARG312	ionic	2.84
					N58	OD2-ASP349	ionic	3.82
11		21	ZINC17160093	-18.99	N42	OD2-ASP349	ionic	3.42
					N47	OD1-ASP214	ionic	2.54
					N47	OD2-ASP349	ionic	3.83
					O51	NE-ARG312	ionic	2.46
					O51	NH1-ARG312	ionic	3.88
					O51	NH2-ARG312	ionic	3.25
					6-ring	CD-ARG312	H-π	4.47
12		23	ZINC00625908	-18.88	N16	OD2-ASP349	HBD	3.38
					N57	OE1-GLU276	HBD	3.06
					N5	OE1-GLU276	ionic	3.60
					N5	OE2-GLU276	ionic	3.74
					N16	OD1-ASP349	ionic	3.76
					N16	OD2-ASP349	ionic	3.38
					N57	OE1-GLU276	ionic	3.06
					N57	OE2-GLU276	ionic	2.99

13	24	ZINC08429954	-18.87	O26	NH1-ARG439	HBA	3.05
				O41	NE-ARG312	ionic	2.96
				O41	NH1-ARG312	ionic	3.97
				O41	NH2-ARG312	ionic	2.88
14	25	ZINC00711743	-18.86	O35	OD1-ASP214	HBD	2.96
15	29	ZINC08441316	-18.66	N57	OD1-ASP214	HBD	2.67
				N57	OD1-ASP214	ionic	2.67
				N57	OD2-ASP214	ionic	3.62
16	30	ZINC04473388	-18.57	N49	O-HOH1228	HBD	2.52
				N28	O-HOH1056	HBA	3.20
				N51	OD1-ASP349	ionic	3.46
				N51	OD2-ASP349	ionic	2.76
				6-ring	6-ring-PHE157	π-π	3.80
17	31	ZINC08411329	-18.48	N50	OD1-ASP349	HBD	2.05
				O10	O-HOH1174	HBA	2.74
				N32	O-HOH1056	HBA	2.32
				N50	OD1-ASP349	ionic	2.05
				N50	OD2-ASP349	ionic	2.61
18	32	ZINC08440549	-18.46	O38	NE-ARG312	ionic	3.33
19	33	ZINC08437172	-18.42	O44	NH1-ARG212	HBA	2.97
				O44	NH2-ARG212	HBA	2.94
				O13	NH1-ARG439	ionic	3.80
				N52	OD1-ASP214	ionic	3.77
20	34	ZINC08441930	-18.35	N61	OD2-ASP214	HBD	2.73
				N61	OE1-GLU276	HBD	2.56
				N61	OH-THR215	HBD	2.16
				N52	O-HOH1026	HBA	2.51
				N59	OD1-ASP68	ionic	2.98
				N59	OD2-ASP68	ionic	3.00
				N61	OD1-ASP214	ionic	2.58
				N61	OD2-ASP214	ionic	2.73
				N61	OE1-GLU276	ionic	2.56
				N61	OE2-GLU276	ionic	3.65
21	36	ZINC08438652	-18.30	O51	O-HOH1174	HBA	2.18
				O49	NE-ARG312	ionic	2.37
				O49	NH2-ARG312	ionic	3.16
				N60	OD2-ASP68	ionic	3.61
22	37	ZINC08441928	-18.29	N61	OE2-GLU276	HBD	2.61
				N59	OD1-ASP68	ionic	3.65
				N59	OD2-ASP68	ionic	1.53
				N61	OD1-ASP214	ionic	3.36
				N61	OE1-GLU276	ionic	2.24
				N61	OE2-GLU276	ionic	2.61
				N61	OD2-ASP349	ionic	3.75
23	38	ZINC08432474	-18.28	O10	O-HOH1102	HBA	3.23
				O23	NE-ARG312	HBA	2.37
				N38	OD1-ASP349	ionic	2.51
				N38	OD2-ASP349	ionic	2.29
24	42	ZINC17160093	-18.14	O24	NH2-ARG312	ionic	3.63
				N34	OD1-ASP349	ionic	3.81
				O49	NE-ARG312	ionic	2.21
				O49	NH1-ARG312	ionic	3.02
				O49	NH2-ARG312	ionic	2.59
				N50	OD2-ASP68	ionic	3.86
				N50	OD1-ASP214	ionic	3.56
25	43	ZINC05313283	-18.14	O13	NE-ARG312	HBA	2.96
				N5	OD1-ASP408	ionic	3.22
				N5	OD2-ASP408	ionic	3.61
				N49	OD2-ASP68	ionic	3.14
				N51	OD1-ASP349	ionic	3.83
				N51	OD1-ASP408	ionic	3.71
26	44	ZINC08438660	-18.05	N58	OD2-ASP349	HBD	2.40
				O57	NE-ARG312	ionic	3.32
				O57	NH2-ARG312	ionic	3.82
				N58	OD1-ASP349	ionic	2.68
				N58	OD2-ASP349	ionic	2.40
27	45	ZINC08438544	-18.05	O25	NE-ARG312	ionic	3.22
				O25	NH1-ARG312	ionic	3.36
				N54	OD2-ASP349	ionic	3.74
				6-ring	CD-ARG312	H-π	4.20
28	46	ZINC08437141	-18.04	N12	OD1-ASP349	HBD	2.59
				O26	OD1-ASP214	HBD	2.31
				O26	NH2-ARG212	HBA	2.66
				N12	OD1-ASP349	ionic	2.59
				N12	OD2-ASP349	ionic	2.79

				6-ring	6-ring-PHE157	π-π	3.49
29	48	ZINC08430547	-18.01	N58	OD1-ASP214	HBD	2.67
				O51	NH1-ARG439	HBA	2.94
				O51	O-HOH1026	HBA	2.17
				N58	OD1-ASP214	ionic	2.67
				N58	OD2-ASP214	ionic	3.22
30	51	ZINC00625938	-17.94	N28	OG1-THR215	HBD	2.65
				N63	OE1-GLU276	HBD	2.22
				O49	O-HOH1026	HBA	2.55
				N28	OE1-GLU276	ionic	2.71
				N28	OE2-GLU276	ionic	3.21
				N47	OD1-ASP214	ionic	3.38
				N47	OD2-ASP349	ionic	3.14
				N63	OD1-ASP214	ionic	3.56
				N63	OE1-GLU276	ionic	2.22
				N63	OE2-GLU276	ionic	2.69
				6-ring	O-HOH1174	H-π	3.43
31	53	ZINC00625757	-17.91	N60	OG1-THR215	HBD	2.31
				N44	OD1-ASP214	ionic	3.62
				N44	OD2-ASP214	ionic	3.51
				N60	OE1-GLU276	ionic	3.91
32	54	ZINC00146770	-17.91	N30	OD2-ASP349	HBD	2.61
				S22	NH1-ARG439	HBA	2.76
				O24	O-HOH1102	HBA	2.88
				N30	OE2-GLU276	ionic	3.45
				N30	OD1-ASP349	ionic	3.44
				N30	OD2-ASP349	ionic	2.61
33	57	ZINC08438548	-17.89	O16	NE-ARG312	ionic	3.47
				O16	NH1-ARG312	ionic	3.32
				O16	NH2-ARG312	ionic	3.68
				O62	NE-ARG312	ionic	2.32
				O62	NH1-ARG312	ionic	3.34
				O62	NH2-ARG312	ionic	1.62
				N63	OD1-ASP349	ionic	3.66
34	58	ZINC00626525	-17.89	O33	NE-ARG439	ionic	3.06
				O33	NH1-ARG439	ionic	1.98
				O33	NH2-ARG439	ionic	3.12
				6-ring	6-ring-PHE157	π-π	3.45
35	59	ZINC08431434	-17.85	N40	OE1-GLU276	HBD	2.52
				N35	OD1-ASP214	ionic	3.86
				N40	OD1-ASP214	ionic	2.61
				N40	OD2-ASP214	ionic	3.57
				N40	OE1-GLU276	ionic	2.52
				N40	OE2-GLU276	ionic	3.25
				6-ring	CD-ARG312	H-π	3.52
36	60	ZINC08411476	-17.83	N42	OD1-ASP349	HBD	2.71
				N26	O-HOH1228	HBA	2.50
				N30	NE-ARG312	HBA	2.57
				N42	OD1-ASP349	ionic	2.71
				N42	OD2-ASP349	ionic	3.04
37	61	ZINC05313297	-17.82	N10	OD1-ASP68	HBD	3.38
38	62	ZINC08437656	-17.81	N21	O-HOH1174	HBA	2.67
				N42	OE2-GLU276	ionic	2.86
				N42	OD2-ASP349	ionic	3.70
39	64	ZINC09070021	-17.76	N59	OE2-GLU276	HBD	3.23
				N59	OE1-GLU276	ionic	3.30
				N59	OE2-GLU276	ionic	3.23
				N59	OD2-ASP349	ionic	3.49
				6-ring	6-ring-PHE157	π-π	3.55
40	65	ZINC02159737	-17.73	N31	O-HOH1102	HBD	1.90
				N56	OD2-ASP349	ionic	3.59
				6-ring	CE1-HIS239	H-π	4.24
41	67	ZINC08438546	-17.60	N54	OD2-ASP214	HBD	2.78
				O24	NH1-ARG439	HBA	2.66
				N54	OD1-ASP214	ionic	2.96
				N54	OD2-ASP214	ionic	2.78
				N54	OE1-GLU276	ionic	3.66
42	70	ZINC06144562	-17.56	N15	OD1-ASP349	ionic	3.58
				N15	OD2-ASP349	ionic	3.70
43	71	ZINC01413496	-17.56	O57	O-HOH1174	HBA	2.12
				O24	NE-ARG312	ionic	2.88
				O24	NH2-ARG312	ionic	3.56
44	72	ZINC00703045	-17.54	O42	NE-ARG439	ionic	3.17
				O42	NH1-ARG439	ionic	3.01
45	74	ZINC06904335	-17.53	N22	OD2-ASP214	HBD	3.56

				O18	NH1-ARG439	ionic	3.33
46	75	ZINC08437908	-17.52	N46	OE1-GLU276	HBD	3.25
				N46	OE1-GLU276	ionic	3.25
				N46	OE2-GLU276	ionic	2.92
47	76	ZINC00702446	-17.51	N37	OD1-ASP214	HBD	2.38
				N32	OD1-ASP349	ionic	3.67
				N32	OD2-ASP349	ionic	3.55
				N37	OD1-ASP214	ionic	2.38
				N37	OD2-ASP214	ionic	3.39
				N37	OE1-GLU276	ionic	3.41
48	77	ZINC08437778	-17.50	N46	OD1-ASP214	HBD	3.04
				N46	OD2-ASP214	HBD	3.21
				O20	NH1-ARG439	HBA	2.84
				N46	OD1-ASP214	ionic	3.04
				N46	OD2-ASP214	ionic	3.21
49	78	ZINC08435576	-17.47	N55	OD1-ASP349	HBD	2.94
				O13	O-HOH1174	HBA	2.54
				O54	NE-ARG312	ionic	2.71
				N55	OD1-ASP349	ionic	2.94
				N55	OD2-ASP349	ionic	2.98
50	79	ZINC08438656	-17.45	O36	NE2-HIS348	HBA	3.19
				O43	NE-ARG312	ionic	2.86
				O43	NH1-ARG312	ionic	3.59
				O43	NH2-ARG312	ionic	2.98
				N57	OD1-ASP349	ionic	2.62
				N57	OD2-ASP349	ionic	3.03
51	82	ZINC00626165	-17.30	N57	OE1-GLU276	HBD	3.12
				O12	NE2-HIS348	HBA	3.12
				O12	O-HOH1026	HBA	2.79
				N5	OE2-GLU276	ionic	3.81
				N5	OD2-ASP349	ionic	3.13
				N57	OE1-GLU276	ionic	3.12
				N57	OE2-GLU276	ionic	2.64
52	83	ZINC00702307	-17.29	O27	NE-ARG312	ionic	2.83
				O27	NH1-ARG312	ionic	3.65
				O27	NH2-ARG312	ionic	3.64
				O54	NE-ARG312	ionic	3.85
53	84	ZINC08438550	-17.28	O62	NE-ARG312	ionic	3.02
				O62	NH2-ARG312	ionic	3.72
				N63	OD2-ASP349	ionic	3.30
54	86	ZINC08432903	-17.23	N57	OE2-GLU276	HBD	2.56
				N57	OE1-GLU276	ionic	3.66
				N57	OE2-GLU276	ionic	2.56
				N57	OD2-ASP349	ionic	3.48
				6-ring	CE1-HIS239	H-π	3.57
55	87	ZINC00703050	-17.23	O34	O-HOH1056	HBA	2.27
				6-ring	CD-ARG312	H-π	3.80
56	89	ZINC08440218	-17.22	N48	OD1-ASP214	HBD	3.04
				N43	OD1-ASP349	ionic	3.07
				N43	OD2-ASP349	ionic	3.16
				N48	OD1-ASP214	ionic	3.04
				N48	OE1-GLU276	ionic	3.65
				N48	OD2-ASP349	ionic	3.63
				O52	NE-ARG312	ionic	2.92
				O52	NH2-ARG312	ionic	3.61
57	90	ZINC08432475	-17.19	N38	OE1-GLU276	HBD	2.87
				N38	OD2-ASP349	HBD	3.44
				O23	O-HOH1056	HBA	2.53
				N38	OD1-ASP214	ionic	3.38
				N38	OE1-GLU276	ionic	2.87
				N38	OE2-GLU276	ionic	3.06
				N38	OD2-ASP349	ionic	3.44
58	91	ZINC08442014	-17.19	N58	OE2-GLU304	HBD	3.24
				O33	O-HOH1026	HBA	2.87
				N25	OE2-GLU276	ionic	3.86
				N25	OD2-ASP349	ionic	3.56
59	92	ZINC00625761	-17.18	N60	OD1-ASP349	HBD	3.05
				N60	OD1-ASP349	ionic	3.05
				N60	OD2-ASP349	ionic	3.49
				6-ring	O-HOH1102	H-π	3.70
60	95	ZINC17160093	-17.11	N51	OD1-ASP214	HBD	1.95
				N35	OD2-ASP68	ionic	3.51
				N51	OD1-ASP214	ionic	1.95
				N51	OD2-ASP214	ionic	2.48
61	96	ZINC08435575	-17.05	N56	OD1-ASP349	HBD	3.75

				O35	O-HOH1056	HBA	2.46
				N56	OD1-ASP349	ionic	3.75
62	97	ZINC00857151	-17.05	N16	OD1-ASP408	HBD	3.36
				O13	O-HOH1174	HBA	2.46
63	99	ZINC00983605	-16.99	N14	OD2-ASP214	HBD	1.97
				N14	OG1-THR215	HBA	1.68
				O17	N-THR215	HBA	2.01
64	100	ZINC15952849	-16.99	O28	NE-ARG439	ionic	3.26
				O28	NH1-ARG439	ionic	3.13
65	101	ZINC08437196	-16.98	N26	OD2-ASP349	HBD	3.29
				N23	OD1-ASP214	ionic	3.34
				N23	OD2-ASP214	ionic	3.03
				N26	OE1-GLU276	ionic	3.53
				N26	OE2-GLU276	ionic	3.23
				N26	OD2-ASP349	ionic	3.29
66	102	ZINC00703108	-16.96	O22	O-HOH1026	HBA	2.53
				O30	NE-ARG439	ionic	2.90
				O30	NH1-ARG439	ionic	2.85
				O32	NH2-ARG312	ionic	3.72
67	104	ZINC08435580	-16.95	O48	NE-ARG312	ionic	3.74
				N60	OD2-ASP349	ionic	3.83
				6-ring	6-ring-PHE157	π-π	3.76
68	105	ZINC02159741	-16.95	O41	NH1-ARG439	HBA	2.70
				O41	O-HOH1026	HBA	2.73
				N49	OD1-ASP214	ionic	3.47
				N49	OD2-ASP214	ionic	3.67
				N49	OE1-GLU276	ionic	3.87
69	106	ZINC08417536	-16.94	O40	NE2-HIS279	ionic	3.49
70	107	ZINC09008533	-16.94	O26	NE-ARG439	ionic	3.22
				O26	NH1-ARG439	ionic	2.38
				O26	NH2-ARG439	ionic	3.28
				O44	NE2-HIS279	ionic	3.62
71	108	ZINC08441623	-16.83	---			
72	109	ZINC08441892	-16.92	N64	OD1-ASP349	HBD	2.38
				N29	NE2-HIS348	HBA	3.03
				N62	OE1-GLU276	ionic	3.99
				N64	OD1-ASP349	ionic	2.38
				N64	OD2-ASP349	ionic	3.52
73	111	ZINC08437653	-16.88	N53	OE1-GLU276	HBD	2.82
				O46	NE2-HIS348	HBA	2.44
				N53	OD1-ASP214	ionic	2.56
				N53	OD2-ASP214	ionic	3.49
				N53	OE1-GLU276	ionic	2.82
				N53	OE2-GLU276	ionic	3.52
74	112	ZINC08996455	-16.86	N62	OD1-ASP349	HBD	3.22
				N62	OD2-ASP349	HBD	2.92
				N62	OD1-ASP349	ionic	3.22
				N62	OD2-ASP349	ionic	2.92
75	113	ZINC00832890	-16.86	N36	O-HOH1228	HBD	2.73
76	117	ZINC06144561	-16.83	N44	O-HOH1228	HBD	2.19
				O43	O-HOH1174	HBA	2.36
				N15	OD2-ASP349	ionic	3.99
77	120	ZINC08435579	-16.80	O40	O-HOH1026	HBA	2.43
				N61	OD1-ASP214	ionic	3.93
				N61	OD2-ASP214	ionic	3.86
78	123	ZINC00625713	-16.77	N66	OE1-GLU276	HBD	2.27
				O52	NE2-HIS348	HBA	3.20
				O52	O-HOH1026	HBA	2.88
				N31	OE1-GLU276	ionic	3.43
				N31	OE2-GLU276	ionic	3.36
				N50	OD1-ASP214	ionic	3.24
				N50	OE1-GLU276	ionic	3.69
				N50	OD2-ASP349	ionic	3.58
				N66	OD2-ASP214	ionic	3.98
				N66	OE1-GLU276	ionic	2.27
				N66	OE2-GLU276	ionic	2.61
79	124	ZINC08441947	-16.77	N57	OD1-ASP349	ionic	3.49
80	125	ZINC08897821	-16.76	N28	OD1-ASP349	ionic	3.17
				N28	OD2-ASP349	ionic	2.86
				N33	OD1-ASP68	ionic	3.56
				N33	OD1-ASP349	ionic	3.26
				O37	NE-ARG312	ionic	2.30
				O37	NH2-ARG312	ionic	3.18
				5-ring	CD-ARG312	H-π	3.74
				5-ring	NE2-HIS348	H-π	3.80

81	127	ZINC09046651	16.71	O26	O-HOH1174	HBA	2.06
				O44	NE2-HIS348	HBA	2.85
				N51	OD1-ASP214	ionic	3.63
				N51	OD2-ASP214	ionic	3.86
				6-ring	6-ring-PHE157	π-π	3.84
82	129	ZINC08432386	16.70	N22	O-HOH1174	HBA	2.71
				O45	NE-ARG312	ionic	2.28
				O45	NH2-ARG312	ionic	3.49
83	130	ZINC17163315	-16.69	O11	NH1-ARG439	HBA	3.11
				O11	O-HOH1026	HBA	2.57
				O24	O-HOH1174	HBA	2.73
84	131	ZINC00711757	-16.69	O14	OD2-ASP68	HBD	2.56
85	132	ZINC08440772	-16.66	O27	NE-ARG439	ionic	1.57
				O27	NH1-ARG439	ionic	0.80
				O27	NH2-ARG439	ionic	1.92
				O27	NH2-ARG443	ionic	3.51
				O50	NE-ARG439	ionic	3.65
				O50	NH1-ARG439	ionic	3.63
				5-ring	6-ring-PHE177	π-π	3.89
86	133	ZINC08441928	-16.65	N53	OE1-GLU276	HBD	2.65
				N53	NH1-ARG212	HBA	3.56
				N61	OD1-ASP68	ionic	2.81
				N61	OD2-ASP68	ionic	2.73
87	134	ZINC20032743	-16.63	N22	OD1-ASP349	HBD	2.93
				N26	OD1-ASP349	HBD	3.37
				N26	OD1-ASP349	ionic	3.37
				N26	OD2-ASP349	ionic	3.08
88	135	ZINC08414138	-16.62	N74	OE2-GLU276	HBD	3.50
				N74	OE2-GLU276	ionic	3.50
89	137	ZINC08897821	-16.61	O34	O-HOH1058	HBA	3.18
				O30	NE-ARG312	ionic	2.81
				O30	NH2-ARG312	ionic	2.75
				N45	OD1-ASP214	ionic	3.42
				N52	OD2-ASP68	ionic	3.39
				N52	OD1-ASP214	ionic	3.60
90	138	ZINC01413485	-16.57	O38	NE-ARG439	ionic	3.64
				O38	NH1-ARG439	ionic	3.61
				O40	NE-ARG439	ionic	3.43
91	139	ZINC08411504	-16.55	N30	NE-ARG312	HBA	3.64
				N46	OD1-ASP349	ionic	2.90
				N46	OD2-ASP349	ionic	2.93
92	140	ZINC05286115	-16.55	N37	O-HOH1102	HBD	2.44
				N39	OE1-GLU276	HBD	2.00
				N5	OD1-ASP408	ionic	3.57
				N32	OD1-ASP214	ionic	2.59
				N32	OD2-ASP214	ionic	3.31
				N39	OD1-ASP214	ionic	2.57
				N39	OD2-ASP214	ionic	3.00
				N39	OE1-GLU276	ionic	2.00
				N39	OE2-GLU276	ionic	3.18
93	141	ZINC08433219	-16.52	N67	OD2-ASP349	HBD	2.95
				O57	O-HOH1026	HBA	2.21
				N67	OD1-ASP349	ionic	3.50
				N67	OD2-ASP349	ionic	2.95
				6-ring	CE2-PHE300	H-π	3.66
94	143	ZINC00683816	-16.47	N34	OD1-ASP214	HBD	1.74
				N34	OD2-ASP214	HBD	1.95
				N38	OD2-ASP68	HBD	1.76
				S37	NE2-HIS111	HBA	2.03
				O14	O-HOH1174	HBA	1.93
				O33	O-HOH1102	HBA	2.46
95	144	ZINC00292755	-16.46	N37	OE2-GLU276	ionic	2.87
96	145	ZINC01414766	-16.46	O31	NE-ARG312	HBA	3.13
				O27	NH1-ARG439	ionic	3.65
				O27	NH2-ARG439	ionic	3.49
				O29	NE-ARG439	ionic	2.46
				O29	NH1-ARG439	ionic	3.42
97	146	ZINC06444803	-16.46	N5	OD2-ASP349	ionic	3.62
				N16	NH1-ARG439	ionic	3.87
				N53	OD2-ASP349	ionic	3.77
				6-ring	5-ring HIS239	π-π	3.71
98	147	ZINC08427102	-16.45	N53	OE1-GLU276	HBD	2.28
				N53	OD1-ASP214	ionic	3.70
				N53	OD2-ASP214	ionic	3.73
				N53	OE1-GLU276	ionic	2.28

				N53	OE2-GLU276	ionic	2.75
				N55	OD1-ASP68	ionic	2.12
				N55	OD2-ASP68	ionic	1.02
99	148	ZINC17160090	-16.41	O21	O-HOH1056	HBA	2.69
				O50	NE-ARG439	ionic	1.79
				O50	NH1-ARG439	ionic	1.30
				O50	NH2-ARG439	ionic	2.57
				5-ring	CB-ARG312	H-π	3.79
100	149	ZINC08438781	-16.40	O13	NE-ARG312	ionic	3.41
				O51	NE-ARG312	ionic	2.59
				O51	NH1-ARG312	ionic	3.60
				O51	NH2-ARG312	ionic	2.37
				N52	OE2-GLU276	ionic	3.75
				N52	OD1-ASP349	ionic	2.81
				N52	OD2-ASP349	ionic	2.02
101	150	ZINC08439872	-16.39	N44	OD2-ASP349	HBD	2.35
				N5	OD1-ASP349	ionic	3.23
				N5	OD2-ASP349	ionic	2.93
				N44	OD1-ASP349	ionic	2.94
				N44	OD2-ASP349	ionic	2.35
102	152	ZINC19989886	-16.36	S33	N-ARG312	HBA	2.82
				N8	OD1-ASP349	ionic	3.82
				N8	OD2-ASP349	ionic	3.68
103	153	ZINC08441926	-16.36	N61	OE2-GLU276	HBD	3.15
				N50	NE2-HIS348	HBA	2.16
				N50	O-HOH1026	HBA	2.77
				N52	O-HOH1087	HBA	2.08
				N59	OD1-ASP68	ionic	3.83
				N59	OD2-ASP68	ionic	2.27
				N61	OD1-ASP214	ionic	2.61
				N61	OD2-ASP214	ionic	3.42
				N61	OE1-GLU276	ionic	2.31
				N61	OE2-GLU276	ionic	3.15
104	154	ZINC08432384	-16.36	N21	O-HOH1174	HBD	1.87
				N55	OD1-ASP408	HBD	3.43
				N55	O-HOH1228	HBD	2.46
				O10	ND2-ASN241	HBA	2.66
				N55	OD1-ASP408	ionic	3.43
105	157	ZINC08414555	-16.34	N58	O-HOH1102	HBD	2.44
				O29	NE-ARG312	HBA	3.04
				N58	OD1-ASP349	ionic	2.39
				N58	OD2-ASP349	ionic	2.41
106	159	ZINC00646487	-16.31	---			
107	160	ZINC09046768	-16.31	O58	NE2-HIS348	HBA	2.30
				N65	OD1-ASP214	ionic	2.99
				N65	OD2-ASP214	ionic	3.58
				N65	OE1-GLU276	ionic	3.89
108	162	ZINC00692735	-16.30	O36	NH2-ARG212	HBA	3.57
				O51	NE2-HIS279	ionic	3.53
				O52	ND1-HIS279	ionic	3.71
				O52	NE2-HIS279	ionic	3.25
109	163	ZINC02162053	-16.29	O28	OD1-ASP349	HBD	2.80
110	165	ZINC08441462	-16.28	O47	O-HOH1056	HBA	2.35
				O45	NH2-ARG312	ionic	3.76
111	166	ZINC08437911	-16.28	N33	OE2-GLU276	ionic	2.86
				N33	OD2-ASP349	ionic	3.41
112	167	ZINC01795564	-16.27	O26	OE1-GLU276	HBD	1.64
				O26	OE2-GLU276	HBD	1.68
				O26	NH2-ARG212	HBA	1.64
				N15	OD1-ASP349	ionic	3.58
				N15	OD2-ASP349	ionic	3.22
113	168	ZINC01413497	-16.27	N34	OD2-ASP349	HBD	2.69
				O57	NE-ARG312	ionic	3.22
				O57	NH1-ARG312	ionic	3.49
				O57	NH2-ARG312	ionic	3.57
114	169	ZINC08429897	-16.26	N22	O-HOH1228	HBD	2.94
				N42	OD2-ASP68	ionic	3.58
				N42	OD2-ASP214	ionic	3.94
115	171	ZINC08437218	-16.24	N15	O-HOH1228	HBD	2.03
				N23	OD1-ASP349	HBD	3.27
				O18	O-HOH1174	HBA	2.61
				N23	OD1-ASP349	ionic	3.27
				6-ring	6-ring-PHE157	π-π	3.62
116	172	ZINC00665548	-16.24	---			
117	173	ZINC08437199	-16.24	O52	NE-ARG312	ionic	1.66

				O52	NH1-ARG312	ionic	2.39
				O52	NH2-ARG312	ionic	2.18
118	174	ZINC00702987	-16.22	---			
119	175	ZINC00630492	-16.21	---			
120	177	ZINC08441601	-16.18	---			
121	178	ZINC02067899	-16.18	O14	O-HOH1228	HBD	1.82
				S24	NE-ARG312	ionic	3.15
				S24	NH1-ARG312	ionic	2.07
				S24	NH2-ARG312	ionic	2.17
122	179	ZINC08430546	-16.17	O44	NE2-HIS348	HBA	2.19
				N51	OD1-ASP349	ionic	3.68
				N51	OD2-ASP349	ionic	3.21
123	180	ZINC08431354	-16.17	N53	OE1-GLU276	HBD	3.32
				N53	OE1-GLU276	ionic	3.32
				N53	OE2-GLU276	ionic	3.17
124	181	ZINC00984875	-16.15	N28	OD2-ASP349	HBD	2.77
				O8	O-HOH1056	HBA	2.44
				O27	NE2-HIS348	HBA	3.13
125	182	ZINC02479986	-16.12	N16	O-HOH1228	HBD	2.37
				N46	OE2-GLU276	ionic	3.92
				N46	OD2-ASP349	ionic	3.45
126	183	ZINC08441602	-16.12	O38	NE-ARG312	ionic	3.00
				O38	NH2-ARG312	ionic	3.82
127	184	ZINC00702427	-16.11	O25	NE-ARG312	HBA	2.53
				O27	NE-ARG439	ionic	2.95
				O27	NH1-ARG439	ionic	3.84
				O56	NE-ARG439	ionic	3.60
				O56	NH1-ARG439	ionic	3.12
				O56	NH2-ARG439	ionic	3.59
128	185	ZINC08414444	-16.11	N62	OD1-ASP349	HBD	3.46
				N62	OD1-ASP349	ionic	3.46
				N62	OD2-ASP349	ionic	3.59
129	186	ZINC06144543	-16.08	N12	OD1-ASP349	HBD	2.46
				O26	NH2-ARG212	HBA	2.14
				N12	OD1-ASP349	ionic	2.46
				N12	OD2-ASP349	ionic	2.76
130	187	ZINC08411455	-16.08	N47	OE2-GLU276	HBD	2.33
				N45	OD2-ASP68	ionic	3.42
				N47	OE1-GLU276	ionic	3.50
				N47	OE2-GLU276	ionic	2.33
131	188	ZINC08429898	-16.06	N42	OD1-ASP214	ionic	2.90
				N42	OD2-ASP349	ionic	3.65
132	190	ZINC01952505	-16.05	N18	NE-ARG439	ionic	3.33
				N18	NH1-ARG439	ionic	3.01
				N20	OD1-ASP349	ionic	3.30
				N40	OD1-ASP349	ionic	3.43
				N40	OD2-ASP349	ionic	3.36
133	191	ZINC08433001	-16.05	N58	OD1-ASP214	HBD	3.39
				O39	NH1-ARG439	HBA	2.06
				N58	OD1-ASP214	ionic	3.39
134	192	ZINC08425651	-16.04	N47	OD1-ASP349	HBD	1.75
				N29	O-HOH1026	HBA	2.27
				N45	OE2-GLU276	ionic	3.66
				N47	OD1-ASP349	ionic	1.75
				N47	OD2-ASP349	ionic	3.29
135	194	ZINC05919492	-16.02	N42	OE2-GLU276	HBD	2.27
				N44	OD2-ASP68	HBD	1.92
				N28	NE2-HIS348	HBA	2.81
				N42	OE1-GLU276	ionic	3.47
				N42	OE2-GLU276	ionic	3.18
				N44	OD1-ASP68	ionic	3.72
				N44	OD2-ASP68	ionic	2.94
136	195	ZINC08436971	-16.01	N53	OD2-ASP68	ionic	3.77
				N53	OD1-ASP214	ionic	3.82
137	196	ZINC08817408	-15.99	N47	OD1-ASP214	HBD	2.52
				N47	OD1-ASP214	ionic	2.52
				N47	OD2-ASP214	ionic	3.66
				C5	6-ring TYR71	H-Π	3.05
138	197	ZINC08411308	-15.98	N36	NE2-HIS348	HBA	3.74
				N52	OD1-ASP349	ionic	2.09
				N52	OD2-ASP349	ionic	2.51
				6-ring	O-HOH1174	H-π	3.80
139	199	ZINC08427099	-15.96	N47	OD1-ASP349	HBD	2.15
				N47	OD1-ASP349	ionic	2.15

				N47	OD2-ASP349	ionic	2.89
140	200	ZINC08437082	-15.95	N11	OD2-ASP349	HBD	2.00
				N15	OD1-ASP214	HBD	1.59
				O22	OD1-ASP214	HBD	2.40
				O22	OD2-ASP214	HBD	1.46
				O14	NH2-ARG212	HBA	1.97
				O14	NE2-HIS348	HBA	2.17
141	201	ZINC08437193	-15.93	N23	OD2-ASP214	ionic	3.61
				N23	OE1-GLU276	ionic	3.27
				N23	OE2-GLU276	ionic	3.98
				C53	6-ring-PHE177	H-π	3.88
				5-ring	O-HOH1102	H-π	3.87
				6-ring	5-ring HIS279	π-π	3.59
142	202	ZINC08417802	-15.93	N39	OD2-ASP349	HBD	2.43
				O38	NE2-HIS348	HBA	2.50
				6-ring	N-ARG312	H-π	4.70

LA = Ligand atom, RA = Receptor atom, HBD = Hydrogen Bond Donor, HBA = Hydrogen Bond Acceptor

Table S3. 3D-structures of selected (eight) Hits.

S. No.	3D-Structures
**1**	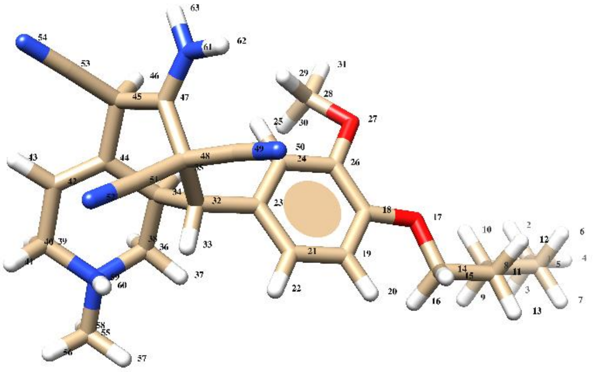 **Compound 20**
**2**	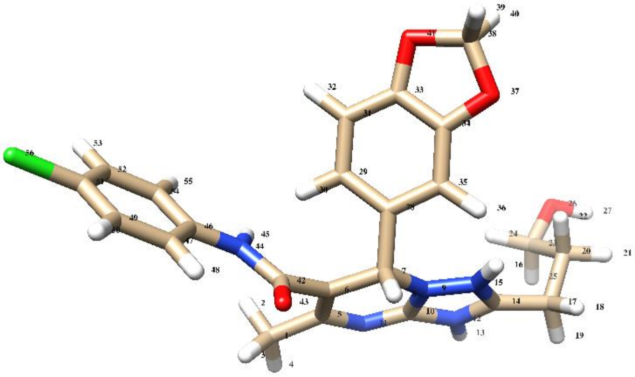 **Compound 28**
**3**	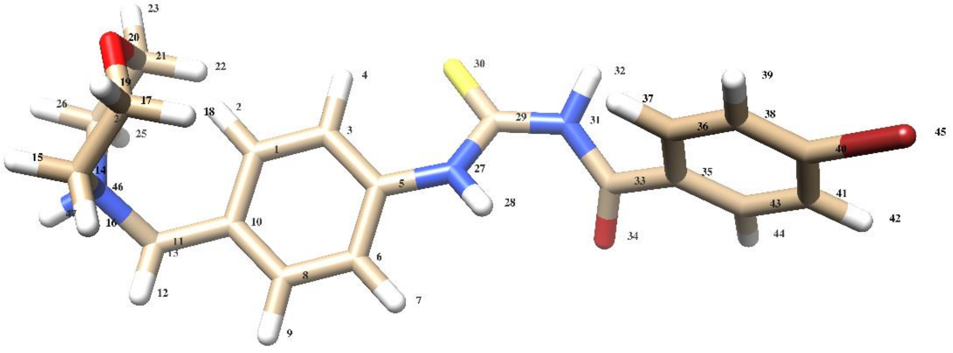 **Compound 48**
**4**	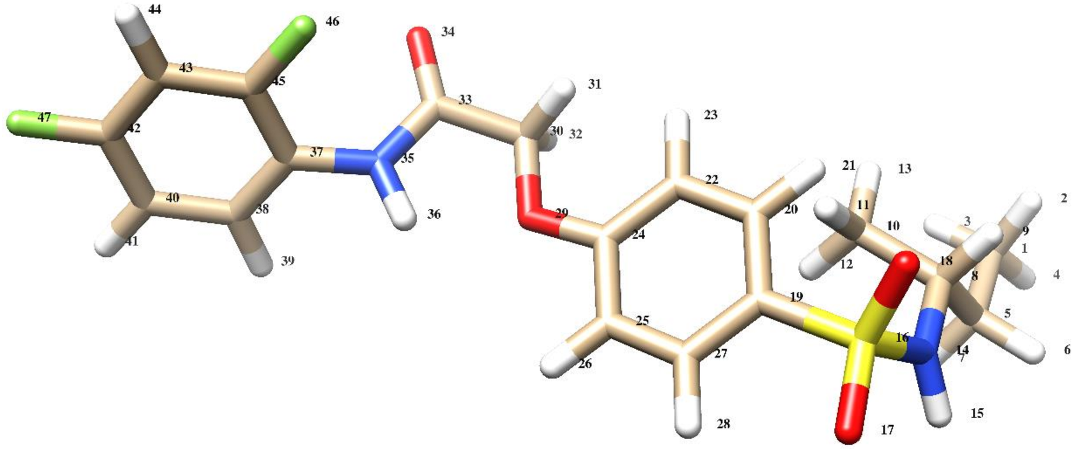

	**Compound 63**
**5**	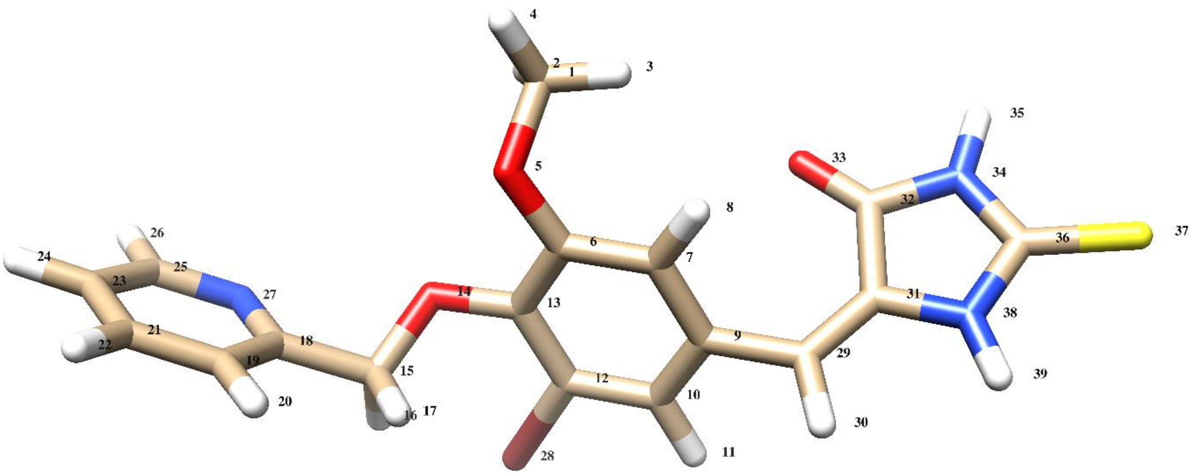 **Compound 94**
**6**	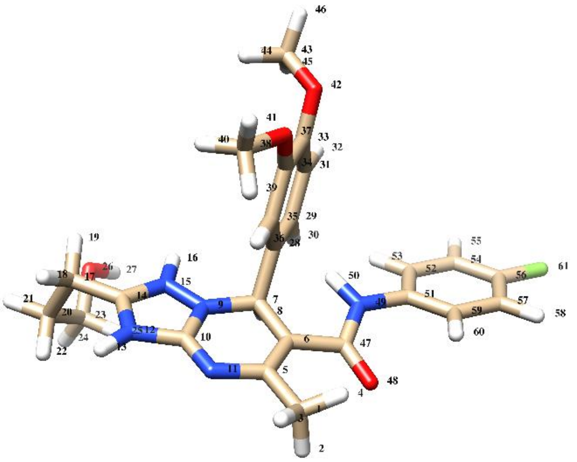 **Compound 112**
**7**	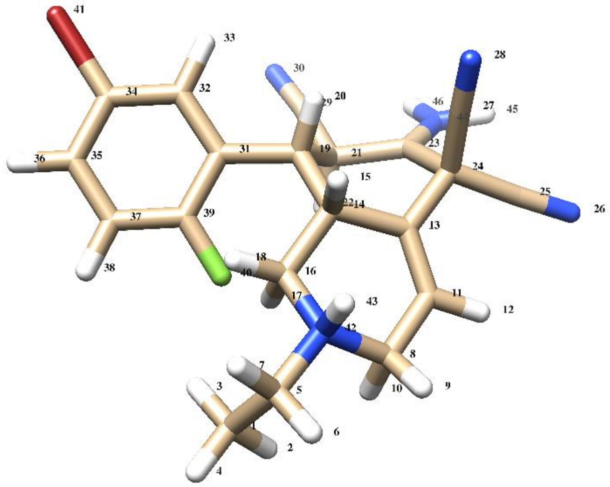 **Compound 135**
**8**	

	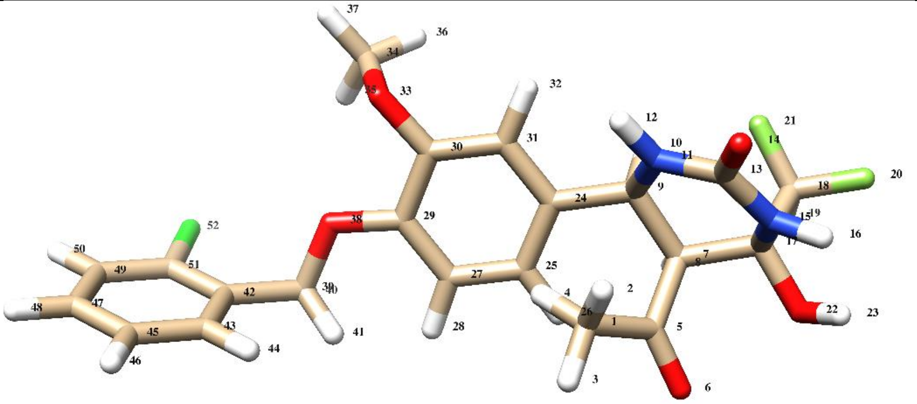 **Compound 140**

Table S4. The predicted activities of thirty-two compounds (Test set 2)

S. No.	Compounds codes in Article	Structure	IC_{50} ± SEM (µM)	pIC_{50}	Predicted Activity	Residual	Reference
1	**3b**	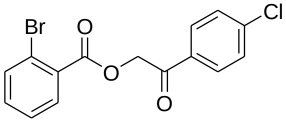	381.7	3.42	3.21	0.21	I. Khan, A. Khan, S.A. Halim, et al., Utilization of the common functional groups in bioactive molecules: Exploring dual inhibitory potential and computational analysis of keto esters against α-glucosidase and carbonic anhydrase-II enzymes, International Journal of Biological Macromolecules (2018), https://doi.org/ 10.1016/j.ijbiomac.2020.11.170
2	**3c**	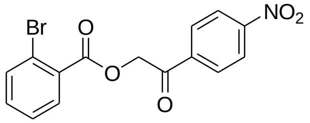	12.4	4.91	4.68	0.23	
3	**3d**	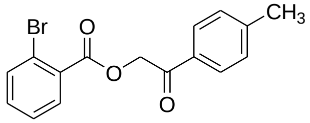	76.5	4.12	4.00	0.12	
4	**3e**	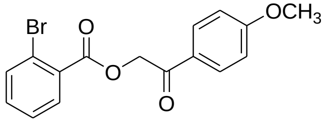	112.4	3.95	3.70	0.25	
5	**3f**	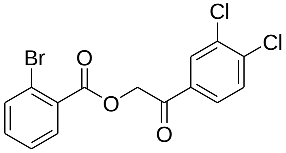	28.0	4.55	4.25	0.30	
6	**3g**	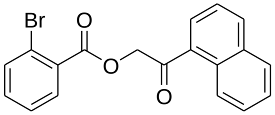	34.1	4.47	4.37	0.10	
7	**3h**	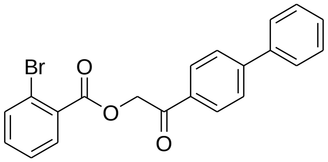	33.9	4.47	4.95	-0.48	

8	**3i**	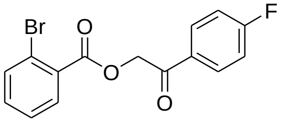	168.8	3.77	3.98	-0.21	
9	**3**	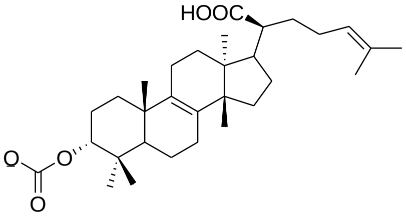	56.8	4.24	4.10	0.14	N. Ur Rahman, S. A. Halim, M. Al-Azri et al., Triterpenic Acids as Non-Competitive α-Glucosidase Inhibitors from Boswellia elongata with Structure-Activity Relationship: In Vitro and In Silico Studies, Biomolecules (2020), https://doi.org/10.3390/biom10050751
10	**4**	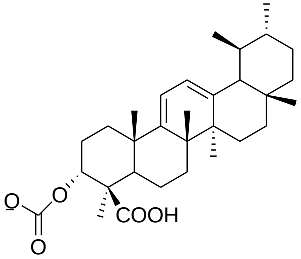	9.9	5.00	5.01	-0.01	
11	**5**	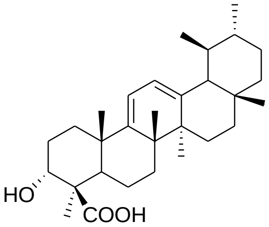	20.9	4.68	4.29	0.39	
12	**11**	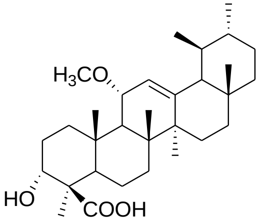	14.9	4.83	4.91	-0.08	

13	**1**	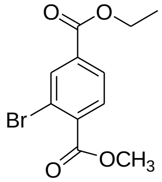	522.0	3.28	3.18	0.10	N. Ur Rahman, K. Rafiq, A. Khan et al., α-Glucosidase Inhibition and Molecular Docking Studies of Natural Brominated Metabolites from Marine Macro Brown Alga Dictyopteris hoytii, Marine Drugs (2019), doi:10.3390/md17120666
14	**2**	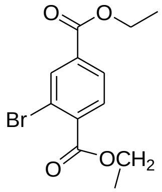	234.2	3.63	3.60	0.03	
15	**3**	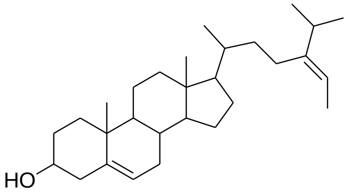	289.4	3.54	3.69	-0.15	
16	**6**	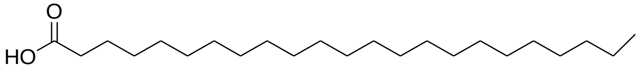	659.78	3.18	3.50	-0.32	
17	**7**	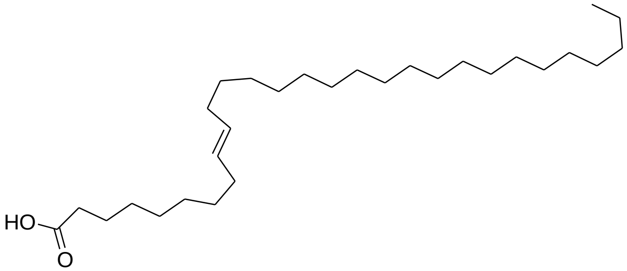	30.5	4.52	4.19	0.33	
18	**8**	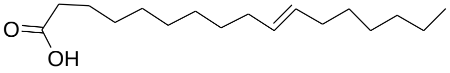	480.1	3.32	3.82	-0.50	

19	**8a**	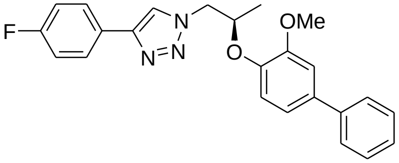	193.7	3.71	4.17	-0.46	S. K. Avula, A. Khan, S. A. Halim, et al., Synthesis of novel (R)-4-fluorophenyl-1H-1,2,3-triazoles: A new class of α-glucosidase inhibitors, Bioorganic Chemistry (2019) https://doi.org/10.1016/j.bioorg.2019.103182
20	**8b**	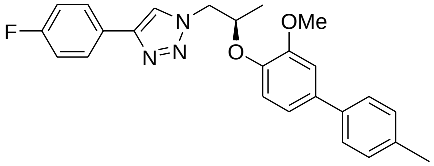	84.2	4.07	4.56	-0.49	
21	**8c**	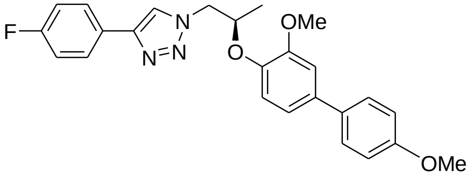	66.8	4.17	4.58	-0.41	
22	**8d**	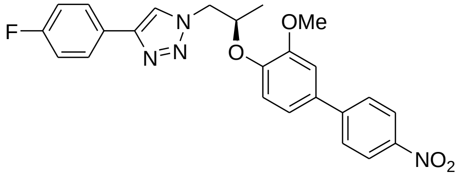	59.5	4.23	4.00	0.23	
23	**8e**	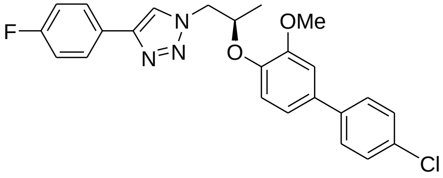	48.6	4.31	3.78	0.53	
24	**8f**	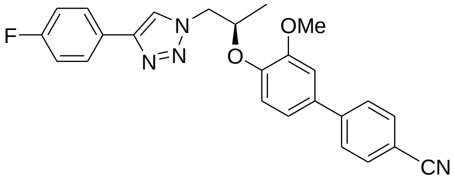	43.7	4.36	4.91	-0.55	

25	**8g**	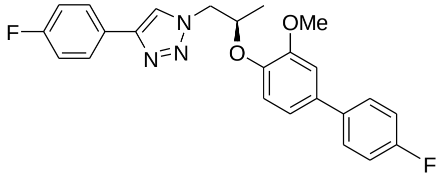	37.6	4.42	4.33	0.09	
26	**8h**	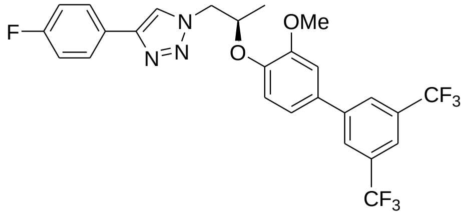	40.18	4.40	4.15	0.25	
27	**8i**	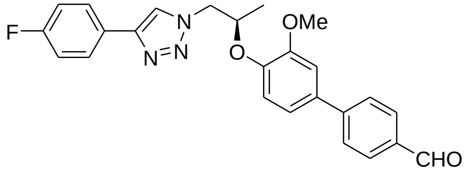	57.0	4.24	4.79	-0.55	
28	**8j**	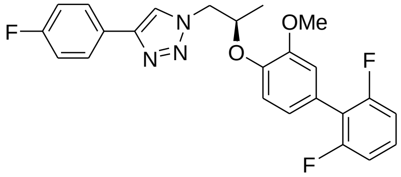	44.3	4.35	3.99	0.36	
29	**8k**	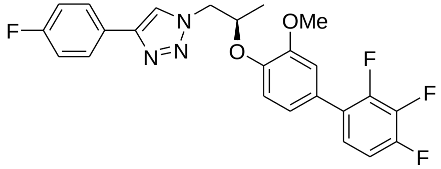	21.7	4.66	4.78	-0.12	
30	**8l**	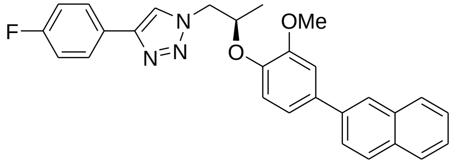	17.9	4.75	4.32	0.43	

31	**8m**	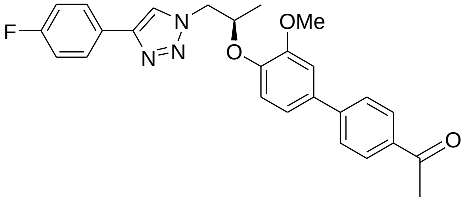	29.5	4.53	4.56	-0.03	
32	**8n**	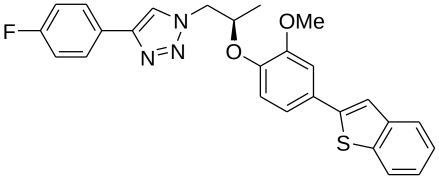	23.9	4.62	5.01	-0.39	

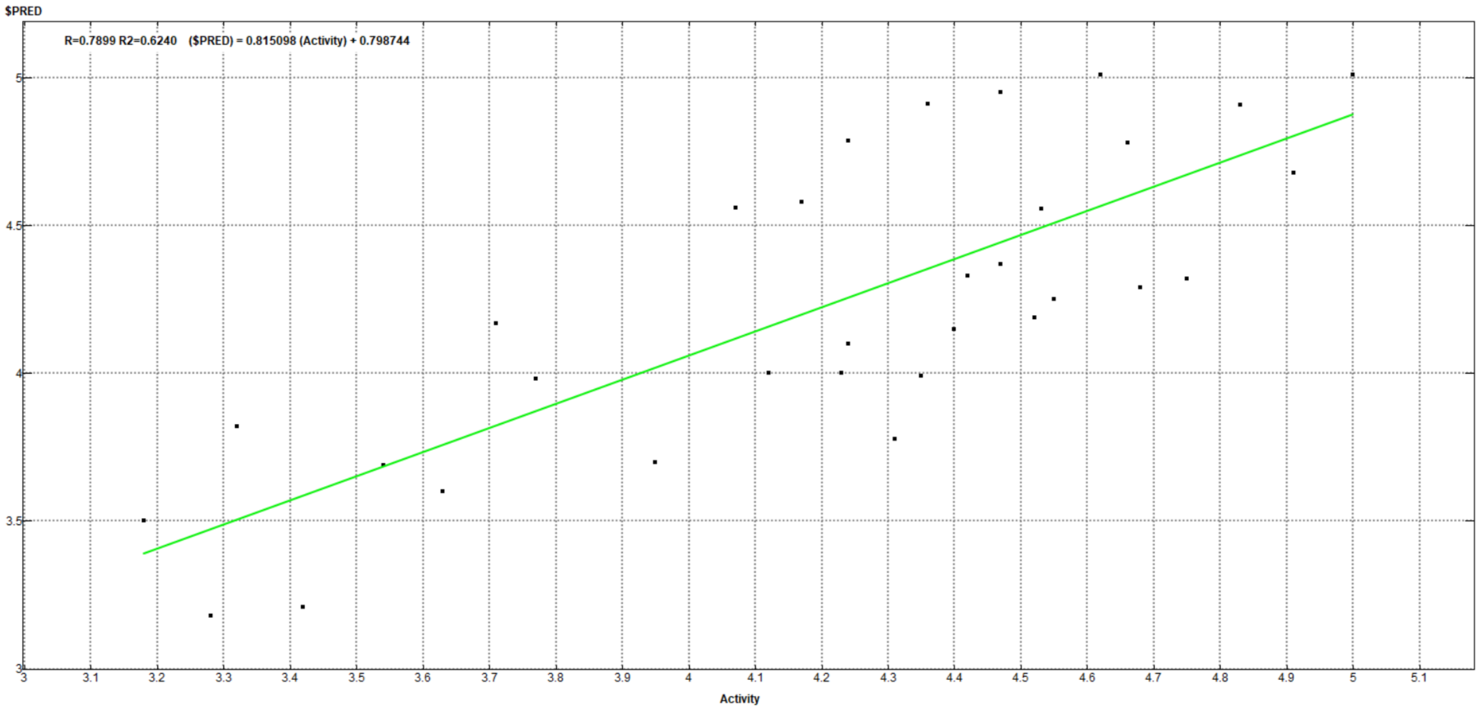

Figure S4. The correlation between Actual activities (pIC_{50}) and Predicted activities ($PRED) of Test set 2 compounds
